# Local experience of laboratory activities in a BS physical therapy course: integrating sEMG and kinematics technology with active learning across six cohorts

**DOI:** 10.3389/fneur.2024.1377222

**Published:** 2024-04-25

**Authors:** Carlos De la Fuente, Alejandro Neira, Álvaro S. Machado, Mauricio Delgado-Bravo, Marcos R. Kunzler, André Gustavo P. de Andrade, Felipe P. Carpes

**Affiliations:** ^1^Exercise and Rehabilitation Sciences Institute, Postgraduate, Faculty of Rehabilitation Sciences, Universidad Andres Bello, Santiago, Chile; ^2^Escuela de Kinesiología, Facultad de Medicina y Ciencias de la Salud, Universidad Mayor, Santiago, Chile; ^3^Laboratory of Neuromechanics, Universidade Federal do Pampa, Uruguaiana, RS, Brazil; ^4^Human Performance Laboratory, Faculty of Kinesiology, University of Calgary, Calgary, AB, Canada; ^5^Carrera de Kinesiología, Departamento de Ciencias de la Salud, Facultad de Medicina, Pontificia Universidad Católica de Chile, Santiago, Chile; ^6^Departamento de Esportes, Escola de Educaçao Física, Fisioterapía e Terapía Ocupacional, EEFFTO-UFMG, Universidade Federal do Minas Gerais, Belo Horizonte, MG, Brazil

**Keywords:** biomechanics, laboratory, active methods, education, sEMG

## Abstract

**Introduction:**

Integrating technology and active learning methods into Laboratory activities would be a transformative educational experience to familiarize physical therapy (PT) students with STEM backgrounds and STEM-based new technologies. However, PT students struggle with technology and feel comfortable memorizing under expositive lectures. Thus, we described the difficulties, uncertainties, and advances observed by faculties on students and the perceptions about learning, satisfaction, and grades of students after implementing laboratory activities in a PT undergraduate course, which integrated surface-electromyography (sEMG) and kinematic technology combined with active learning methods.

**Methods:**

Six cohorts of PT students (*n* = 482) of a second-year PT course were included. The course had expositive lectures and seven laboratory activities. Students interpreted the evidence and addressed different motor control problems related to daily life movements. The difficulties, uncertainties, and advances observed by faculties on students, as well as the students’ perceptions about learning, satisfaction with the course activities, and grades of students, were described.

**Results:**

The number of students indicating that the methodology was “always” or “almost always,” promoting creative, analytical, or critical thinking was 70.5% [61.0–88.0%]. Satisfaction with the whole course was 97.0% [93.0–98.0%]. Laboratory grades were linearly associated to course grades with a regression coefficient of 0.53 and 0.43 R-squared (*p* < 0.001).

**Conclusion:**

Integrating sEMG and kinematics technology with active learning into laboratory activities enhances students’ engagement and understanding of human movement. This approach holds promises to improve teaching-learning processes, which were observed consistently across the cohorts of students.

## 1 Introduction

Integrating neurophysiology, motor control, and neuromechanics of human movement can be challenging for undergraduate health students due to the concepts that they are required to learn and understand in academic curricula like physical therapy (PT) (1–3). The non-stationary nature of neurophysiology (4), i.e., the rapid fluctuations of voltage created by ion fluxes in plasmatic membranes, and the lack of practice in critical thinking and reasoning, make motor control and neuromechanics difficult for PT students. PT curricula might be related to this limitation because of their topographical approach, memorized skill, and passive teaching focus within the first years of 5-years long programs (5, 6). However, this can be overcome using demonstrations (7), practical lessons, and active learning methodologies, which involve the student learning rather than relegating them to a passive role as observers (8, 9). In addition, surface electromyography (sEMG) or kinematics technology may facilitate the learning of students (8) during demonstrative practices (9) because these bioinstruments can show real-time instantaneous changes that PT students can actively interact with, and analyze.

Although there are several choices for measuring sEMG, the dry bipolar sEMG allows the quantification of the sum of trains of motor units action potential voltages produced by muscle fibers when contracted. Dry bipolar sEMG has low time consumption and can reproduce motor patterns under controlled conditions for learning purposes in PT (10, 11). The sEMG with this aim has been called kinesiological sEMG (12, 13). This sEMG has been widely and safely applied non-invasively (12–14). For kinesiological assumptions, it is relevant to complement the sEMG measures with synchronized kinematics to understand the action of muscles during the movement. sEMG is not an out of reach technical challenges (12, 13, 15, 16), but its implementation for teaching can be found on www.robertomerletti.it, the YouTube channel NeuromechTV, the BiomeCast (17), or ISEK-JEK tutorials (8, 18–20).

Combining sEMG and kinematics with active learning methods in practical activities would encourage PT students to learn, analyze, and discuss actively under a critical thinking and science context. Critical thinking “is the ability to raise discriminating questions to search for better ideas, a deeper understanding, and better solutions relating to a given issue” (21), and science involves the use of the scientific method. Such skills are crucial to criticizing data, identifying whether conclusions are supported by evidence, and distinguishing the effects of an intervention or stimulus (22). Health students can elaborate more sophisticated reasoning (22) and improve their professional practice when using critical thinking (21). Thus, to explore the difficulties (the level of challenge that learners experience during the learning process (23)), uncertainties [the cognitive impasse while understanding something, resulting in uncertainty, lack of clarity, disorientation, contradiction or mental pause (23, 24)], and advances [educational important improvements similarly to clinical importance meaning (25)] of students perceived by faculties is essential to improve laboratory activities for PT student. This may fill the gap related to familiarity with science, technology, engineering and mathematics (STEM) background and the STEM-based new technologies that can shape the future of rehabilitation (11). Consequently, we aimed to describe the difficulties, uncertainties, and advances observed by faculties on students, and the perceptions about learning, satisfaction, and grades of students after implementing laboratory activities in a PT undergraduate course, which integrated sEMG and kinematic technology combined with active learning methods.

## 2 Methods

### 2.1 Experimental design

In this brief report, we retrospectively analyzed six cohorts of second-year PT undergraduate students (PT bachelor that lasts five years) with a mixed study design (qualitative and quantitative study) between 2013 and 2018 as a local initiative in a university ranked 301–400 in life sciences and as the best Latin American university in the Time Higher Education Ranking 2023. The students were assigned to a semester PT course (Table 1) delivered with expositive classes (focused on functional anatomy, joint anatomy, joint surface movement, and neuromechanics) every week and seven laboratory activities across the semester (18 weeks). Laboratory activities were programmed after a theoretical expositive class. The students solved seven motor problems, one for each laboratory activity incorporating sEMG and kinematic devices under active learning methods (see the teaching methodology section for details). The problems were designed based on daily life movements (see the Motor task section in the Supplementary material: Methods for details).

**Table 1:** Course modules and topics.

No. of Class	Time of teaching (hrs)	Who taught?	Module	Author reference	Topics
1	1.5	Laboratory	Introduction to Human Movement	M. Latash J Athl Train. 2002; 37(1): 80–84. J Athl Train. 2002;37(1):71–79.	• Neurophysiology of Human Movement ○ Proprioception and joint stability ○ Sensorimotor integration ○ Reflexes ○ Preprogrammed responses
2	3	Laboratory	Introduction to Human Movement	Shumway-Cook & C. Woollacott Curr Opin Neurobiol. 1999;9(6):718–727.	• Motor control theories ○ Equilibrium point hypothesis ○ Trajectory in equilibrium ○ Internal models ○ Computational theory
3	1.5	Laboratory	Introduction to Human Movement	R. Enoka	• Mechanics ○ Degree of freedom and vectorial algebra ○ Equilibrium ○ Kinematics and chains ○ Dynamics
4	1.5	Laboratory	Introduction to Human Movement	G. Robertson R. Enoka R. Merletti & P. Parker	• Bioinstrumentation ○ Analog/digital conversion ○ Resolution ○ Videophotogrametry ○ Accelerometry ○ Force platforms ○ Electromyography
5	3	Laboratory	Biomechanics of tissues	D. Neuman M. Nordin & V. Frankel	• Material resistance introduction ○ Loads, stress, tensors, stiffness, and strain ○ Elasticity, viscosity, and Viscoelasticity ○ Isotropy and anisotropy ○ Elastic and plastic behaviors ○ Ultimate and yield strength, and Fatigue
6	1.5	Clinician	Biomechanics of tissues	D. Neuman M. Nordin & V. Frankel	Biomechanics of Bones
7	1.5	Clinician	Biomechanics of tissues	D. Neuman M. Nordin & V. Frankel	Biomechanics of Nerves
8	1.5	Clinician	Biomechanics of tissues	D. Neuman M. Nordin & V. Frankel	Biomechanics of Ligaments and tendons
9	1.5	Clinician	Biomechanics of tissues	D. Neuman M. Nordin & V. Frankel	Biomechanics of cartilage
10	3	Laboratory	Biomechanics of tissues	D. Neuman M. Nordin & V. Frankel	Biomechanics of skeletal muscle
11	3	Clinician	Biomechanics of Joints	D. Neuman	• Clinical Introduction to Joint Biomechanics ○ Torque ○ Plane and axis ○ Joint surface and joint kinematics ○ Kinematic chain
12	3	Clinician	Biomechanics of Joints	D. Neuman	• Shoulder joint complex ○ Anatomy ○ Joint surface kinematics ○ Scapular-humeral rhythm ○ Coaptation of the shoulder ○ Ligament and Muscles stabilizers
13	3	Clinician	Biomechanics of Joints	D. Neuman	• Elbow joint complex ○ Anatomy ○ Joint surface kinematics ○ Ligament and Muscles stabilizers
14	3	Clinician	Biomechanics of Joints	D. Neuman	• Forearm and wrist joint complex ○ Anatomy ○ Joint surface kinematics ○ Ligament and Muscles stabilizers ○ Hand grip
15	3	Clinician	Biomechanics of Joints	D. Neuman	• Hand joints ○ Anatomy ○ Joint surface kinematics ○ Ligament and Muscles stabilizers ○ Pinch grips
16	3	Clinician	Biomechanics of Joints	D. Neuman	• Temporo-mandibular Joint ○ Anatomy ○ Joint surface kinematics ○ Ligament and Muscles stabilizers
17	3	Clinician	Biomechanics of Joints	D. Neuman	• Cervical and thoracic spine ○ Anatomy ○ Joint surface kinematics ○ Ligament and Muscles stabilizers
18	3	Clinician	Biomechanics of Joints	D. Neuman	• Lumbar spine and sacrum ○ Anatomy ○ Joint surface kinematics ○ Ligament and Muscles stabilizers
19	3	Laboratory	Biomechanics of Joints	D. Neuman M. Nordin & V. Frankel	• Biomechanics of Gait ○ Kinematics ○ Kinetics ○ Muscle activations patterns ○ Motor control theories
20	3	Clinician	Biomechanics of Joints	D. Neuman M. Nordin & V. Frankel	• Hip joint ○ Anatomy ○ Joint surface kinematics ○ Ligament and Muscles stabilizers ○ Joint reaction forces
21	3	Clinician	Biomechanics of Joints	D. Neuman M. Nordin & V. Frankel	• Knee joint ○ Anatomy ○ Joint surface kinematics ○ Ligament and Muscles stabilizers ○ Joint reaction forces
22	3	Clinician	Biomechanics of Joints	D. Neuman M. Nordin & V. Frankel	• Foot and ankle joints ○ Anatomy ○ Joint surface kinematics ○ Ligament and Muscles stabilizers ○ Extrincic and Intrincic foot muscules ○ Joint reaction forces

All classes were expositive (passive methodology) and distributed over 18 weeks. The author’s references were detailed in the teaching methodology subsection. The Modules and topics were defined by the curricular committee of the PT department.

The laboratory faculties (CD and MD) had a Bachelor degree in PT, clinical experience in musculoskeletal PT, clinical experience in clinical biomechanics, sEMG background, between five and eight years of experience as research undergraduate student assistants, with expertise in topics of sEMG and motor control, both faculties improved their knowledge during their clinical biomechanics MSc program, and one did a Bioengineering MSc receiving engineering training, and also studied an evening/night school engineering program. In addition, they had between 3 and 5 years of experience teaching study topics, with experience in learning methodology, familiarity with biomechanical instrumentation, and digital programming skills. Both faculties completed equipment training from companies for the appropriate use and care of sEMG and kinematic devices.

This study was approved by the local institutional ethics committee and conducted according to the principles of the Declaration of Helsinki. The participants were anonymized, and the university blinded the applied questionnaire without access to the faculties (only general results were allowed for faculties; see the raw results in the Supplementary material: Questionnaires raw results).

### 2.2 Participants

Participants were 482 s-year PT students (2013: *n* = 69, 2014: *n* = 73, 2015: *n* = 74, 2016: *n* = 100, 2017: *n* = 72, and 2018: *n* = 94). These students were between 18 and 22 years old and shared a homogeneous background in health sciences. Admission to the university from high school was based on similar criteria for all students, including grades and national exam results (26). Prior to participating in the study, students had completed foundational courses in histology, anatomy, math, biostatistics, physics, physiology, exercise physiology, and biochemistry (27).

It is noteworthy that none of the PT students had any significant background in technology, particularly in the field of biomechanics. This lack of prior exposure to biomechanics-related concepts or laboratory-using technology settings ensured that all participants started from a similar baseline of knowledge and experience.

### 2.3 Teaching methodology

**Figure 1:**
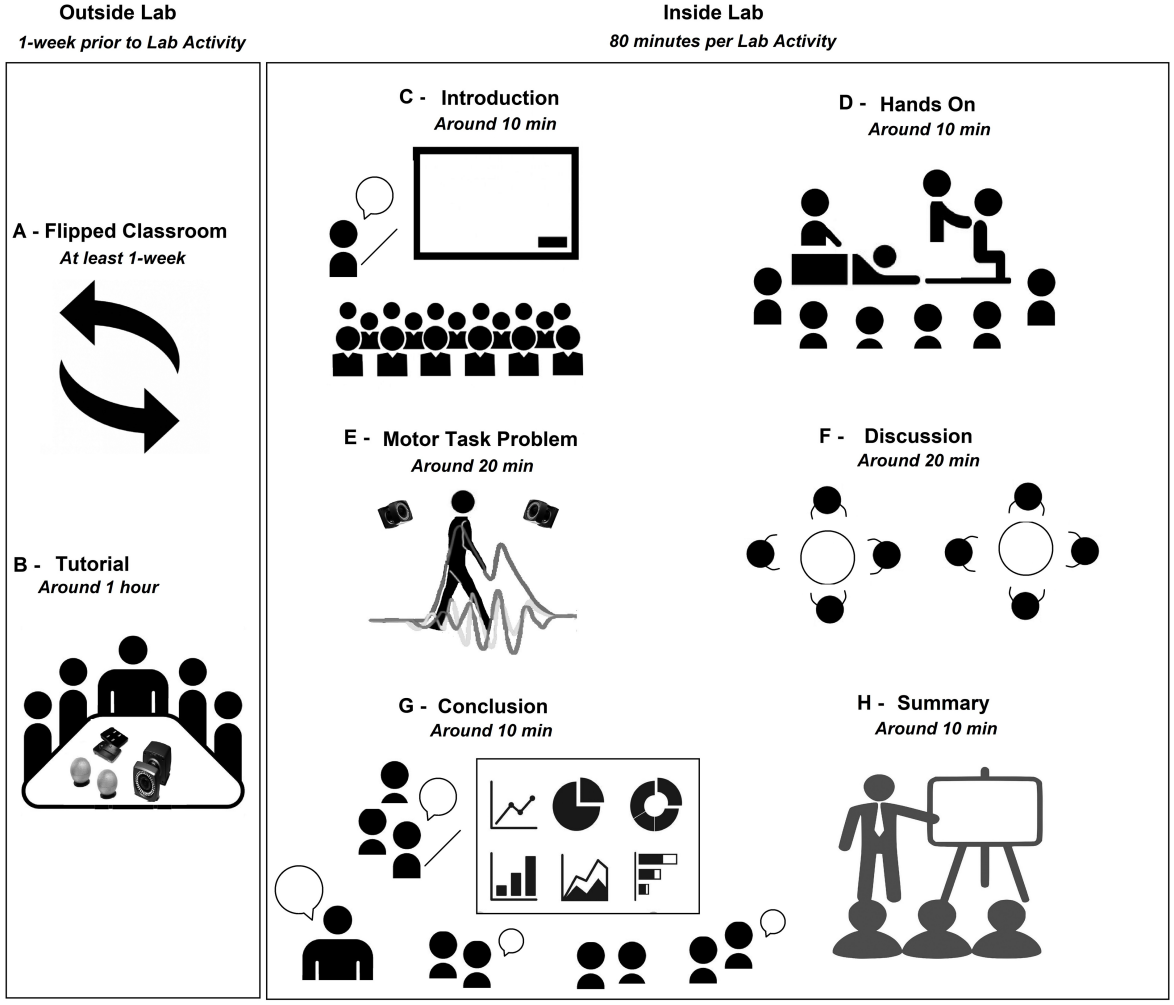
Active learning methodology in the laboratory.

When the academic semester started, the students were self-organized in small groups of 4 to 5, according to their preferences and affinity with a collaborative team philosophy. The small groups were organized into two schedules (two days in two daily schedules) to promote collaborative teamwork and avoid teaching many attendees (we had seven groups with a maximum of 25 attendees in the laboratory). One small group was defined as the leader group for each laboratory activity to present the introduction, the motor task problem to solve, and the methods of the activity (Figure 1). The leader group was hands-on in the measurements of the motor task with the participation of other small groups and the active support of the laboratory faculties.

The students could read and assimilate the contents of the laboratory activity prior to each laboratory activity through a flipped classroom, an active method that replaces teacher-led in-class instructions with individual homework or group activities prior to the lesson (28) (Figure 1A). The students could access the recommended literature list when they joined the course. The recommended literature includes textbooks (“Kinesiology of the Musculoskeletal System of Neuman,” “Motor Control: Translating Research to Practice of Shumway-Cook & Woollacott,” “Basic Biomechanics of the Musculoskeletal System of Nordin & Frankel,” “Neuromechanics of the human movement of Enoka,” “Neurophysiology of the human movement of Latash,” “Research Methods in Biomechanics of Robertson,” and “Electromyography: Physiology, Engineering, and Non-Invasive Applications of Merletti & Parker”) and some scientific papers depending on the topic (see Table 1 and the Motor task section in the Supplementary material: Methods for details). Next, the leader group met with the faculties to discuss the motor task problem and learn about the technology needed to measure the motor task. Also, the sequence of the activities and roles was explained, and the faculties summarized the lecture content of the flipped class and introduced the bio-instruments needed for the activity (Figure 1B).

During the day of the activity, the students were individually assessed through a quiz to score each Laboratory ten minutes before the start of the laboratory activity (see a quiz example for Laboratory activity No. 2 in the Supplementary material: Quiz example). Then, the laboratory activity was conducted by the small leader group (Figure 1C). At the same time, the other groups attended the introduction and methods, actively participated in the hands-on analysis and discussion of the motor task problem, and in the class summary (in total 80 min of activity, See Figures 1D–H). Each session started with the presentation of the introduction, the motor problem task, and methods to solve the assigned problem. Here, the faculties filled in any forgotten content or made corrections, and a PT comment was always linked with the activity. The other groups heard the presentation (around 10 min, Figure 1C). After that, the hands-on activity on a volunteer was developed. Here, the faculties supported the leader group and encouraged all students to participate in the hands-on activities guided by the leader group and faculties, explaining and asking all the students questions about anatomy, neurophysiology, motor control, or neuromechanics. All students surrounded the work area for better visualization (around 10 min, Figure 1D). Then, the faculty collected, processed and shared the data from the motor task problem with all laboratory attendees. Here, the students explored the results of the measures and understood the data to be interpreted during the discussion (around 20 min, Figure 1E). Immediately after, each small group independently discussed the data collection and answered the questions of the activity written in the material given prior to each laboratory activity. The faculties actively helped and guided the understanding of the neuromechanical elements involved in the task problem and encouraged independent critical thinking for each small group. Each small group had to prepare a conclusion to be shared by the end of the activity (around 20 min, Figure 1F). Then, the leader group started sharing their findings and conclusions, followed by the other groups. The faculties encouraged the discussion and questioned conclusions to stimulate critical thinking (around 10 min, Figure 1G). Finally, the faculties summarized and clarified the most important neuromechanical elements/concepts involved in the motor tasks and gave a take-home message for all students (around 10 min, Figure 1H). To ensure the correct technical aspects of the activities, the faculties controlled the timing of the activities, adopted the role of an external observer (contrary to the teacher-center model) to transfer the protagonist role to the students, processed the data to plot the results in real-time, and assisted students with any technical aspects during the laboratory activity. For example, faculties guided the placement of sEMG sensors according to the SENIAM project (29), which was projected on a screen in the laboratory. Moreover, the faculties showed the students the topographical and palpation anatomy on the volunteer. When there were doubts, a human skeleton anatomy model and internet images were used.

### 2.4 Motor tasks

**Figure 2:**
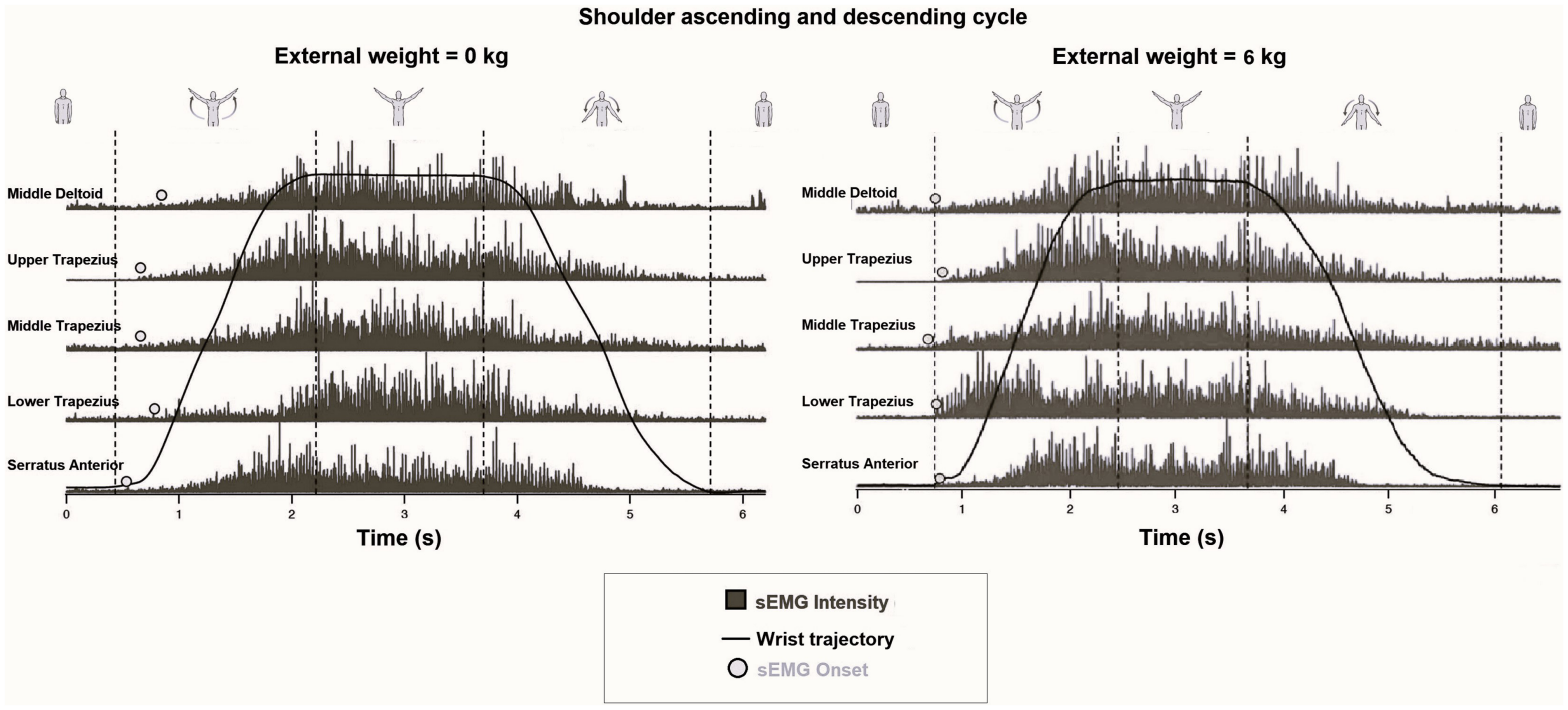
Example of a full rectified sEMG (middle Deltoid, Serratus Anterior, and Upper, Middle, and Lower Trapezius), sEMG onset, wrist position, and different phases of the shoulder abduction (rest, ascending, maintaining, and descending) extracted from shoulder abduction without weight (at left) and with 6 kg in each hand (at right) during the second laboratory activity. It is appreciated that muscle activation shifts to the left for all evaluated muscles when the shoulder elevates 6 kg in each hand against gravity acceleration. The graphs have not been intensity-normalized and the effect of load on muscle activation onset is considered.

Seven motor tasks were proposed based on a problem-based approach and real-life problems (Please see the Motor task section in the Supplementary material: Methods and Figure 2 as example).

### 2.5 Instrumentation

For details on instrumentation, please see the Supplementary material: Methods.

### 2.6 Perception of difficulties, uncertainties, and advances

The two laboratory faculties retrospectively and qualitatively summarized in a text (transcription process) their experience of past teaching laboratory activities laboratory by laboratory. Difficulties, uncertainties, and advances of students in each laboratory activity were enlisted individually (qualitative description). An independent faculty enlisted topics to find themes or patterns from the unstructured data written by the laboratory faculties, including these themes when both faculties agreed upon a consensus. Codes (tags or labels that are assigned) from the explicit content of the data (the list of difficulties, uncertainties, and advances of students in each laboratory observed by each laboratory faculty) were generated. This stage formally ended the qualitative coding process. All themes were determined using inductive coding, which consists of deriving codes from the data. This qualitative approach allows theory to emerge from data, being a discovery and exploratory strategy for unstructured data.

### 2.7 Course learning evaluation

When the course was concluded, each student individually answered a blinded questionnaire administered online by the university administration in which three questions were directly related to the laboratory activities. The first question was “*has the course promoted your creative, analytical, and critical thinking?*,” and the second question was “*how often did the professor link the course content to real-life situations?.*” The students ranked the answers for each of the two questions as “*never*” or “*almost never*,” “*rarely*,” “*many times*,” and “*always*” or “*almost always*.” The third question was “*are you satisfied with this course?*,” and the possible answers were “*yes*” or “*no*.”

The whole questionnaire was created in 1992, and since this year, undergraduate courses at the university have been systematically and blindly (for faculties) evaluated every semester. The assessment tool was validated with high psychometric properties. For details on the questionnaire and psychometric measurement, please visit https://direcciondedesarrolloacademico.uc.cl/academicos/encuesta-docente. The results received by faculties for the whole raw questionnaire were attached as Supplementary material.

### 2.8 Assessment of the course and laboratory activities

The student performance in the laboratory was quantified through the average score in 7 quizzes related to the laboratory activities, resulting in the laboratory grades for each student (Please see a quiz example of the laboratory No.2 Supplementary material of quiz example). The resolution of laboratory and course grades was 1 pts./question and 0.12 pts./question, respectively.

The course performance was quantified by the weighted average from three written tests (45%), a poster presentation in which each group presented a short literature review on the topic of motor tasks addressed in the laboratory meeting (15%), the laboratory grade (10%), and the final written exam (30%). All grades were scored between 1 and 7 pts.

Grades below 4.0 pts. were considered as *failure*, grades 4 as fair, 5 as good, 6 as *very good*, and 7 as *excellent*. At least 4.0 pts. were needed to be approved in the course (60% of difficulty).

### 2.9 Statistical analysis

The perceptions of the faculties about the difficulties, uncertainties, and advances experienced by the students across each laboratory activity were qualitatively described in a summary table (30). Data normality of grades was confirmed for quantitative measurements using the Kolmogorov–Smirnov test. The course learning perceptions and the course and laboratory activities were described as median and [minimal – maximum] over the six cohorts of students. We performed a linear regression to identify how the laboratory grades independently explain the variance of the course grades, obtaining the 95% confidence interval of the linear model. All statistical analyses considered an alpha set at 5%. The calculations were made using GraphPad Prism software 5.0 (GraphPad Prism software, inc., USA).

## 3 Results

The laboratory activities difficulties, uncertainties, and advances observed by the laboratory faculties for each laboratory activity are summarized in Table 2.

**Table 2:** Description of difficulties, uncertainties, and advances of the students during active learning methods using surface electromyography (sEMG) and kinematic technology in PT Laboratory activities.

No.	Motor task problem	Students’ difficulties, uncertainties and advances (educational important improvements)	
1	Bipedal control during expected and non-expected anteroposterior perturbations	Difficulties	Remembering the lower extremity anatomy. Understanding the physiological meaning of raw sEMG signal changes.
		Uncertainties	Differentiating the type of muscle action (eccentric, concentric, isometric). They only learned anatomy based on concentric actions of muscles. Distinguishing between central and peripheral responses of the nervous system (voluntary and involuntary movements).
		Advances	After the laboratory, there was a better global comprehension of ankle muscle actions for postural control responses using sEMG raw signals.
2	Scapular and shoulder control with and without wrist weight while reaching	Difficulties	Remembering the shoulder anatomy. Understanding the kinematics meaning of signal and signal changes.
		Uncertainties	Understanding how the shoulder abduction (scapular and humerus movements) is controlled by each muscle action (eccentric, concentric actions), the interaction of gravitational force, and external and internal torques.
		Advances	After the laboratory, there was an increased understanding of the physiological meaning of sEMG, including the full-rectified method, onset, timing, and main intensity.
3	Elbow and wrist stabilization during the hand grip test changes muscle lengths	Difficulties	Remembering the elbow, forearm, and wrist anatomy. Understanding the muscle mechanics (force-length relation): forearm muscle length changes (three different wrist angles) on the increase and decrease of force (output: handgrip strength).
		Uncertainties	Understanding how muscles cross multiple joints and their mechanical effects in different joints.
		Advances	After the laboratory, the student showed better strategies for analyzing muscle actions. After the laboratory, there was a better interpretation of sEMG intensity, including RMS measurements.
4	Abdominal and spinal stabilization during lifting tasks	Difficulties	Remembering the spine muscles and thoracolumbar fascia anatomy. Understanding accessory and abdominal strategies aimed at enhancing lumbar stabilization through increased intra-abdominal pressure.
		Uncertainties	Understanding the vectorial diagrams of intra-abdominal pressure and lumbar vertebras instability. Understanding the vectorial diagrams of fascia to cause lumbar extension moment.
		Advances	After the laboratory, there was improved integration between muscle activation and mechanical outputs for active and passive spine stabilization mechanisms. After the laboratory, there was an improved qualitative capacity to observe co-activations from raw sEMg signals.
5	Hip control during gait	Difficulties	Identifying the terminology and mechanical functions of joints during gait. Identifying eccentric and concentric actions and their mechanical significance during the gait cycle.
		Uncertainties	Understanding how the nervous system controls the hip during the gait and interacts with gravitational force and external and internal torques. Understanding gait phases and events. Analyzing movements in a plane different than the sagittal plane.
		Advances	After the laboratory, there was an improved understanding of gait terminology and joint mechanics.
6	Knee control during gait	Difficulties	Understanding the roles of muscle actions and their mechanical effect when crossing multiple joints. Some students tend to memorize concepts rather than analyze them (these students advance more slowly).
		Uncertainties	Analyzing movements in a plane different than the sagittal plane.
		Advances	After the laboratory, there were improvements in identifying tasks and phases during gait in the sagittal plane and in interpreting muscle mechanical functions during gait at knee level (damping versus propulsion).
7	Ankle control during gait	Difficulties	Understanding the roles of muscle actions and their mechanical effect when crossing multiple joints. Understanding ankle and foot mechanics. Integrating the whole neuromechanics of the lower limb joints during gait
		Uncertainties	Understanding foot and ankle muscle actions. Analyzing movements in a plane different than the sagittal plane. Understanding of external forces and torques in the foot and ankle.
		Advances	After the laboratory, there was an improvement in understanding the mechanical interpretation of muscle actions during gait at ankle and foot level (damping versus propulsion). After the laboratory, there was an improved capacity to observe co-activations from raw and envelopes sEMg signals.

sEMG, surface electromyography.

**Figure 3:**
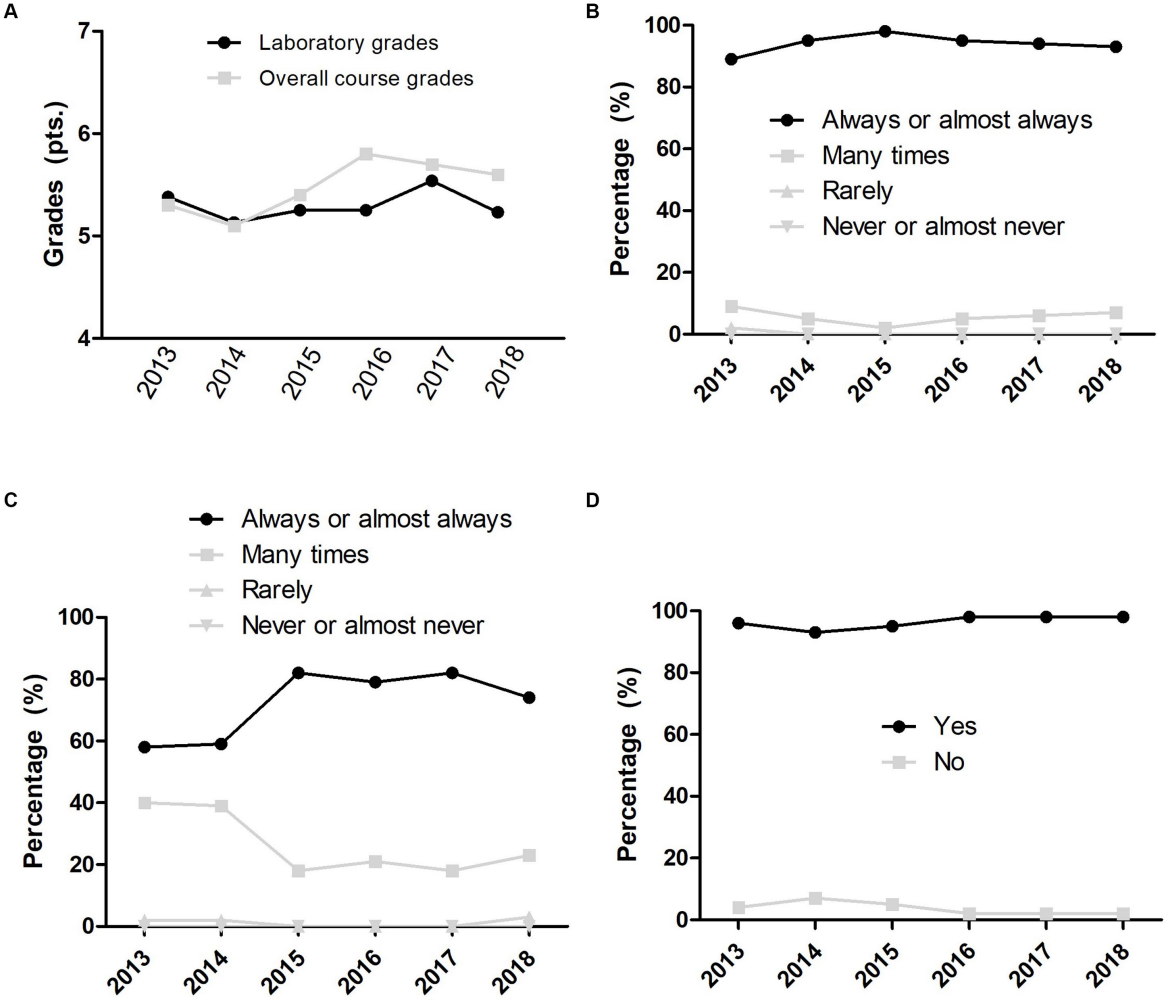
Physical therapy discipline performance from 2013 to 2018. **(A)** Grades. **(B)** Self-report of real-life examples involved in the course. **(C)** Self-report of critical thinking involved in the course. **(D)** Self-perception of satisfaction with the course.

The total of students (*n* = 482) considered that the course “always” or “almost always” promoted their creative, analytical, or critical thinking with a median of 70.5% [61.0% (minimum value) – 88.0% (maximal value)]. The total students (*n* = 482) perceived that the faculties “always” or “almost always” linked the course content to real-life situations with a median of 94.5% [89.0% (minimum value) – 98.0% (maximal value)]. All the students (*n* = 482) felt satisfied with the course development with a median of 97.0% [93.0% (minimum value) – 98.0% (maximal value)]. See Figure 3.

**Figure 4:**
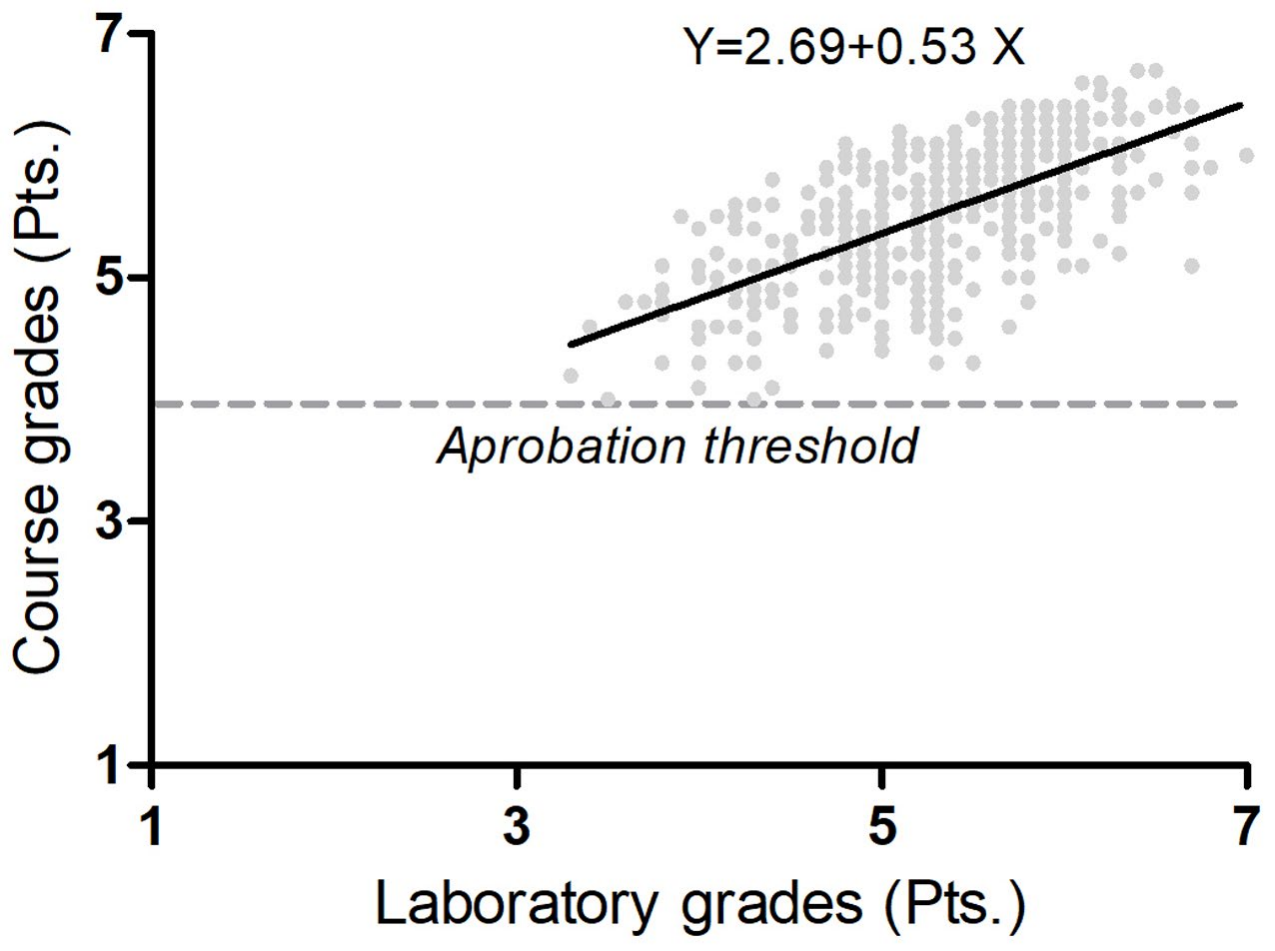
Regression analysis. The figure shows the linear model (black line over samples) that explained with statistical significance (*p* < 0.001) the course grades (*y*-axis) by the laboratory grades (*x*-axis) over 482 students of physical therapy. Grades below 4 were considered as failed, grade 4 as fair, grade 5 as good, grade 6 as very good, and 7 as Excellent. The resolution of grade was 0.1 pts. The course performance was quantified by the weighted average from three written tests (45%), a poster presentation in which each group presented a short literature review on the topic of motor tasks addressed in the laboratory meeting (15%), the laboratory grade (10%), and the final written exam (30%).

The laboratory grades for the total of students (*n* = 482) achieved a median of 5.3 pts. [3.3 pts. (minimum value) – 7.0 pts. (maximal value)]. Moreover, the course grades for the total of students (*n* = 482) achieved a median of 5.6 pts. [4.0 pts. (minimum value) – 6.7 pts. (maximal value)]. The linear regression model obtained was (laboratory grades) = 2.69 + 0.53 (course grade) with 480 degrees of freedom, slope 0.534 [95% confidence interval: 0.479 to 0.590] and Y-intercept 2.691 [95% confidence interval: 2.395 to 2.987], statistical significance (*p* < 0.001), a root mean square error of 0.416, and an R-squared of 0.427 (Figure 4).

## 4 Discussion

The main findings of this study indicate that the laboratory activities: (i) Challenged the students to learn and understand based on the difficulties and uncertainties that were effectively managed and led to improvements in student learning. This finding aligns with effective learning strategies, emphasizing the importance of encountering challenges as integral components of deep learning processes. (ii) The activities promoted critical thinking and facilitated the application of course content to real-life movements, and high levels of satisfaction with the whole course were obtained. This satisfaction trend was consistently observed across six different cohorts. (iii) Additionally, laboratory grades explained 43% of the total variance in the course grades with a moderate association (regression coefficient of 0.53). From the learning point of view, these results are relevant because when PT students started the course, they felt that laboratory activities and technology will not provide helpful knowledge for their future clinical practice and tended to be comfortable memorizing concepts. In this sense, the active methodologies and the addition of sEMG and kinematics in real-time allow PT students to explore new strategies for human movement analysis and develop their critical thinking and reasoning supported by the scientific method, obtaining important learning and attitude changes. The laboratory activities induced students to adopt a critical and causative point of view on why signals changed in front of them. The purpose of our methods was a transformation of PT to engage with STEM backgrounds and the STEM-based new technologies familiarization that can impact the rehabilitation of patients.

The most relevant learning difficulties were the human anatomy background (the PT anatomy is not only the origin and muscle fibers orientation knowledge; also the irrigation, innervation, myotomes, dermatomes, medullar roots of nerves, or detailed bone landmarks were asked), identifying the muscle actions (eccentric, concentric, or isometric), integrating the physics concepts (gravity force, external and internal torques), and identifying the mechanical effects of muscle actions crossing more than one joint. It is suggested that a fresh previous background is more important when the active methodology is used because the students’ active class participation would immediately show their deficits. Due to that, our method might cause a cumulative source of difficulties and uncertainties, especially for those who tried to memorize instead of analyze. Thus, tuning the difficulties and uncertainties, filling gaps in knowledge, and managing (focusing) the study of students in and outside the laboratory are crucial issues to producing effective learning (23, 24). Laboratory activities create the opportunity to uncover the ideas of students as well as the students’ difficulties and uncertainties (31). Our motor task problems challenged the students through collaborative discussions (32) and technology use (6). Previous evidence suggests that active methodologies, such as those we performed, positively impact students’ learning (32) and better address inter-student learning variability, which in PT students has been mainly identified with collaborative learning style (33).

Especially for PT students, the active methods of using sEMG and kinematics measurements permitted them to address better the neuromechanics and motor control topics (34). The promoted critical thinking linked the course content to real-life movements, and reasoning practice was crucial for the early development of human movement analysis skills that may favor understanding the mechanisms behind human movements in agreement with the benefits of incorporating practical lessons in the students’ learning (35, 36). The study of neuromechanics and motor control in PT is a challenge because this is often presented as managed by high-level central structures, neglecting the peripheral role in human movement control (37) or is not considered in musculoskeletal PT because the attention is centered on the morphology and movement of the joint surfaces. In consequence, the combination of different active methods in the laboratory activities where technology can measure instantaneous changes in real-time (briefly: depending on the need, the student must understand how analog signals end up in discretized signals, Nyquist frequency, synchronization, drifting, etc.) linked with real-life movements suggest the need to improve the students learning in agreement with the effects of blended learning methodology based on real-life movements (38) and how laboratory activities improve the theory comprehension of students (31). Also, high satisfaction was observed among the students with the whole course, and the students perceived our activities as applicable and connected to the main course aims, which was consistently observed across six different cohorts.

The linear model used to study the course grades and the laboratory grades as the independent variable fits with an R-squared of 43%, and both variables were associated with a regression coefficient of 0.53 (moderate association). It may be that students who performed well or poorly in the course tend to perform similarly in answering the laboratory quizzes. We suggest that laboratory activities assessment should not only be limited solely to quizzes because it is essential to consider other skills directly related to the laboratory activities to understand better how these activities influence theoretical courses in PT. On the other hand, some students failed the laboratory, demonstrating that the laboratory demanded a higher learning effort (39) than the overall course. The laboratory-based learning effort may develop higher-level thinking skills, such as understanding, analyzing, and evaluating (40), in contrast to the whole course, which mainly focuses on memorization skills. The focus on memorization skills in this course is in coherence with how the PT clinician professors reserve critical thinking skills development only when students perform clinical courses. We suggest that the development of higher-level thinking skills can start during the first PT courses, as we have proposed in this study.

Unfortunately, our teaching has been interrupted due to four main reasons: (i) In 2019, the country suffered political instability affecting university teaching. (ii) From 2020 to 2022, the coronavirus pandemic forced the course into online and hybrid modalities, prompting the reorganization of classes (17). (iii) In 2023, two newly incorporated faculty members opted not to further develop this activity, reverting to passive lectures and reducing laboratory activities. (iv) In our, as well as in other countries, newly nominated professors focus on publications and grant applications over teaching commitments. Additionally, recent graduates joining the faculty lack the knowledge and autonomy to conduct such activity, in agreement with recent reports (11). Hence, effective implementation of laboratory activities in PT require familiarity with biomechanical instrumentation (technical independence), clinical and teaching experience in human movement analysis and rehabilitation, signal processing skills, a track record of publications, grant writing capacity, and familiarity with both active and passive learning methods.

As a limitation, our study only assessed a cohort (without a control group) due to indications of the institution. It gave the same methodologies to all students without chances to make changes if the curriculum did not indicate it. Future directions should improve our laboratory activities, methods, and study design. More shared experiences are needed to reach a better solution to engage PTs with STEM backgrounds and STEM-based new technologies. Research faculties should not be exclusively dedicated to their own research lines. The BS classroom should be an excellent opportunity to prepare students for rehabilitation using technology, doing research and impacting the rehabilitation of patients. Finally, PT departments must introduce technology and measurement techniques to the students involved in rehabilitation and not only focus on teaching manual and clinical skills. Innovative teaching and learning methods and specialized PT familiarized with STEM would be fundamental to this transformation.

## 5 Conclusion

PT students did not initially perceive the value of laboratory activities and technology for their future clinical practice and tended to be comfortable memorizing concepts more than understanding, analyzing, and evaluating them. However, the integration of sEMG and kinematics technology with active learning during PT laboratories engaged students in learning and enhanced their understanding of the link between the neuromuscular system and mechanical elements of human movement. This innovative methodology shows promise for improving teaching-learning processes and presents challenges for both faculty and students. Importantly, these outcomes were consistently observed across six different cohorts of students, demonstrating the robustness and effectiveness of the approach.

## Data availability statement

The original contributions presented in the study are included in the article/Supplementary material, further inquiries can be directed to the corresponding author.

## Ethics statement

The studies involving humans were approved by Universidad Catolica institutional review board. The studies were conducted in accordance with the local legislation and institutional requirements. The participants provided their written informed consent to participate in this study.

## Author contributions

CF: Conceptualization, Data curation, Formal analysis, Investigation, Methodology, Project administration, Resources, Software, Supervision, Validation, Visualization, Writing – original draft, Writing – review & editing. AN: Formal analysis, Funding acquisition, Validation, Writing – original draft, Writing – review & editing. ÁM: Conceptualization, Validation, Writing – review & editing. MD-B: Conceptualization, Validation, Writing – review & editing. MK: Conceptualization, Investigation, Methodology, Validation, Writing – original draft, Writing – review & editing. AA: Conceptualization, Methodology, Validation, Writing – original draft, Writing – review & editing. FC: Conceptualization, Formal analysis, Methodology, Validation, Writing – original draft, Writing – review & editing.
