# Supplementary material for: Local experience of laboratory activities in a BS physical therapy course: integrating sEMG and kinematics technology with active learning across six cohorts
Source: Front Neurol. 2024 Apr 25;15:1377222. doi: 10.3389/fneur.2024.1377222 (PMC11081031; doi:10.3389/fneur.2024.1377222)
Supplement: Supplementary file 1 [file Data_Sheet_1.PDF]

ENCUESTA DE CALIDAD DOCENTE

REPORTE DE RESULTADOS PARA DOCENTES

Para la elaboración de este reporte se utilizaron datos extraídos el 16 de diciembre.

1. DATOS DE IDENTIFICACIÓN

Docente	[redacted]
UA del docente	
N° de docentes curso-secc.	
Tipo asignatura	
Sigla	
Sección - Total secciones	
Curso	
UA del curso	

2. DATOS DE LA EVALUACIÓN

Año y semestre aplicación	2013 - 22
Total de alumnos que completó la encuesta	57
Porcentaje encuestas respondidas en relación a alumnos inscritos	80%

3. CARACTERIZACIÓN ALUMNOS

Total de alumnos inscritos en el curso	71
Promedio Ponderado Acumulado (PPA) del grupo	5,3
Promedio de notas de los alumnos en el curso	5,3
Promedio de créditos aprobados por los alumnos	175,6
Porcentaje alumnos que reportó que el curso contó con ayudantía presencial	53%

ENCUESTA DE CALIDAD DOCENTE

Asistencia a clases - Dedicación fuera de clases - Carácter de inscripción del curso (declarado):

El porcentaje de clases (o actividades clínicas, terreno, laboratorio, etc.) a las que asistí en este curso con este profesor fue:					
**0% - 24%**	**25% - 49%**	**50% - 74%**	**75% - 100%**	**Total respuestas (n)**	**% omisión***
0%	2%	9%	89%	56	2%

¿aproximadamente cuántas horas dedicaste trabajando o estudiando para este curso fuera de las clases presenciales regulares?					
**3 o menos**	**4 a 6 horas**	**7 a 9 horas**	**10 o más**	**Total respuestas (n)**	**% omisión***
30%	42%	25%	4%	57	0%

Considerando la malla de tu carrera, indica en qué calidad tomaste este curso				
**Mínimo**	**Optativo de profundización**	**Optativo de formación general**	**Total respuestas (n)**	**% omisión***
95%	5%	0%	57	0%

*Corresponde al porcentaje de alumnos que no respondió el ítem.

ENCUESTA DE CALIDAD DOCENTE

4. RESULTADOS DE ÍTEMS POR DIMENSIÓN

A continuación se presentan sus resultados por ítem agrupados según las dimensiones de esta evaluación.

- Porcentaje observado en cada opción de respuesta de cada ítem.
- Promedio de cada ítem.
- Desviación estándar de cada ítem. Informa la variabilidad de los puntajes de los participantes, es decir cuán cercanas o dispersas se encuentran las evaluaciones realizadas respecto del puntaje "promedio por ítem". Una desviación estándar de valor cero indicaría que todos los alumnos evaluaron a un profesor con un mismo puntaje; mientras más alto el valor del dato, mayor variabilidad de las respuestas de los estudiantes.
- El total de respuestas que se recibieron en cada ítem.
- El porcentaje de omisión por ítem, que corresponde a la proporción de estudiantes que respondieron la encuesta, pero no respondieron el ítem.

A nivel de cada dimensión reportada además se entrega la siguiente información:

- Porcentaje observado en cada opción de respuesta de los ítems que componen cada dimensión, en base al total de respuestas que recibió dicha dimensión.
- Promedio de cada dimensión, calculado en base al total de respuestas de los alumnos.
- Desviación estándar de cada dimensión, calculado en base al total de respuestas de los alumnos.
- El total de respuestas que se recibieron en cada dimensión.
- El porcentaje de omisión por dimensión, que corresponde a la proporción de estudiantes que respondió la encuesta pero no respondió ningún ítem de los que componen esa dimensión.

Las opciones de respuesta para los ítems corresponden a una escala de 4 puntos, siendo el valor mínimo 1 y el valor máximo 4, a excepción de las preguntas Sí – No del apartado de Apreciación Global (en que los valores máximos y mínimos son 1 y 0, respectivamente).

ENCUESTA DE CALIDAD DOCENTE

4.1. Dimensión Aplicación del conocimiento

	Porcentaje de respuestas por ítem							
**¿Durante este curso (taller o laboratorio), con qué frecuencia el docente...**	**Nunca o casi nunca (1)**	**Solo en pocas clases (2)**	**En varias clases (3)**	**En la mayoría de las clases (4)**	**Prome-dio**	**Desv. están-dar**	**Total resptas. (n)**	**% omisión**
...usó bibliografía o recursos (de información, audiovisuales, artísticos u otros) variados y atingentes los objetivos del curso?	0%	2%	21%	77%	3,8	0,47	57	0%
... vinculó los contenidos del curso a ejemplos asociados a situaciones reales o hipotéticas?	0%	2%	9%	89%	3,9	0,38	57	0%
...presentó nuevas ideas, hallazgos o metodologías asociadas a los contenidos del curso?	0%	7%	35%	58%	3,5	0,63	57	0%
**Total dimensión Aplicación del conocimiento**	0%	4%	22%	75%	3,7	0,38	57	0%

ENCUESTA DE CALIDAD DOCENTE

4.2. Dimensión Organización de la enseñanza

**Con respecto a la organización de la enseñanza en este curso**	**Porcentaje de respuestas por ítem: Nunca o casi nunca (1)**	**Pocas veces (2)**	**Muchas veces (3)**	**Siempre o casi siempre (4)**	**Prome-dio**	**Desv. están-dar**	**Total resptas. (n)**	**% omisión**
Los tiempos dedicados a las distintas actividades del curso fueron adecuados	0%	5%	35%	60%	3,5	0,60	57	0%
Existió una clara relación entre los contenidos del curso y las actividades pedagógicas implementadas (prácticas, laboratorios, reflexiones u otras)	0%	0%	16%	84%	3,8	0,37	57	0%
El desarrollo y secuencia de las clases, talleres o laboratorios fueron claras y facilitaron mi aprendizaje	0%	4%	18%	79%	3,8	0,51	57	0%
**Total de la dimensión Organización de la enseñanza**	0%	3%	23%	74%	3,7	0,40	57	0%

**(este ítem no es considerado en el promedio de la dimensión)**	**Nunca o casi nunca (1)**	**Pocas veces (2)**	**Muchas veces (3)**	**Siempre o casi siempre (4)**	**Prome-dio**	**Desv. están-dar**	**Total resptas. (n)**	**% omisión**
Hubo una adecuada coordinación de la ayudantía con el desarrollo del curso	5%	8%	26%	62%	3,4	0,85	39	32%

ENCUESTA DE CALIDAD DOCENTE

4.3. Dimensión Metodología de enseñanza aprendizaje

	Porcentaje de respuestas por ítem							
**¿Durante este curso (taller o laboratorio), con qué frecuencia el docente estimuló...**	**Nunca o casi nunca (1)**	**Pocas veces (2)**	**Muchas veces (3)**	**Siempre o casi siempre (4)**	**Promedio**	**Desv. estándar**	**Total resptas. (n)**	**% omisión**
… que los estudiantes hicieran preguntas durante las clases?	0%	4%	16%	80%	3,8	0,50	56	2%
... que los estudiantes buscaran información o investigaran en forma autónoma?	0%	18%	39%	43%	3,3	0,74	56	2%
... que los estudiantes trabajaran en grupo en alguna actividad dentro o fuera de la sala de clases?	2%	2%	28%	69%	3,6	0,62	54	5%
**Total dimensión Metodología de enseñanza aprendizaje**	1%	8%	28%	64%	3,5	0,51	56	2%

4.4. Dimensión Evaluación y retroalimentación a los estudiantes

	Porcentaje de respuestas por ítem							
**¿Durante este curso (taller o laboratorio), con qué frecuencia el docente…**	**Nunca o casi nunca (1)**	**Pocas veces (2)**	**Muchas veces (3)**	**Siempre o casi siempre (4)**	**Promedio**	**Desv. estándar**	**Total resptas. (n)**	**% omisión**
… realizó evaluaciones con criterios explícitos y conocidos por todos los estudiantes?	0%	0%	18%	82%	3,8	0,38	57	0%
… entregó a tiempo retroalimentación (individual o grupal) acerca de las fortalezas y debilidades del desempeño de los estudiantes?	0%	9%	28%	63%	3,5	0,66	57	0%
… utilizó diferentes métodos, situaciones, formas o tipos de preguntas para evaluar los aprendizajes del curso?	0%	9%	30%	61%	3,5	0,66	57	0%
**Total dimensión Evaluación y retroalimentación a los estudiantes**	0%	6%	25%	69%	3,6	0,47	57	0%

ENCUESTA DE CALIDAD DOCENTE

4.5. Dimensión Relación con estudiantes

Con respecto de la relación entre docente y estudiantes	Porcentaje de respuestas por ítem				Prome-dio	Desv. están-dar	Total resptas. (n)	% omisión
	Nunca o casi nunca (1)	Pocas veces (2)	Muchas veces (3)	Siempre o casi siempre (4)				
El docente estuvo disponible para responder mis consultas en el horario acordado de atención de los estudiantes	0%	2%	18%	81%	3,8	0,45	57	0%
El docente fue respetuoso con todos los estudiantes del curso	0%	0%	4%	96%	4,0	0,19	56	2%
El docente mostró confianza en la capacidad de aprender de los estudiantes del curso	0%	0%	13%	87%	3,9	0,34	55	4%
**Total dimensión Relación con estudiantes**	0%	1%	11%	88%	3,9	0,28	57	0%

5. ÍTEMS DE APRECIACIÓN GLOBAL

En la siguiente tabla, el porcentaje de respuestas "Sí" corresponde a la proporción de estudiantes que respondieron la encuesta y marcaron esa opción. El porcentaje de omisión, es la proporción de estudiantes que respondieron la encuesta, pero no respondieron el ítem.

	% Sí	% No	Total respuestas (n)	% omisión
¿Recomendarías este profesor a un amigo?	98%	2%	57	0%
¿Quedaste satisfecho con este curso?	96%	4%	57	0%

ENCUESTA DE CALIDAD DOCENTE

6. ÍTEMS DE APRENDIZAJE PERCIBIDO

	Porcentaje de respuestas por ítem							
	Mucho menos de lo espera-do (1)	Menos de los espera-do (2)	Lo espera-do (3)	Mucho más de lo espera-do (4)	Prome-dio	Desv. están-dar	Total resptas. (n)	% omisión
Más allá de la nota que has obtenido, ¿cuánto aprendiste en este curso?	0%	5%	54%	40%	3,4	0,58	57	0%
Este curso promovió mi pensamiento creativo, analítico o crítico	0%	2%	40%	58%	3,6	0,54	57	0%

7. RESUMEN RESULTADOS POR DIMENSIÓN: TOTAL UNIDAD ACADÉMICA

A continuación se presenta un resumen de los puntajes por dimensión para el Total Unidad Académica.

ID: 16-11978406-KIN Id1: 38538

	Porcentaje de respuestas por ítem							
	Nunca o casi nunca (1)	Pocas veces (2)	Muchas veces (3)	Siempre o casi siempre (4)	Prome-dio	Desv. están-dar	Total resptas. (n)	% omisión
Dimensión Aplicación del conocimiento (+)	2%	5%	22%	71%	3,6	0,55	391	0%
Dimensión Organización de la enseñanza	2%	7%	26%	65%	3,6	0,59	391	0%
Dimensión Metodologías enseñanza aprendizaje	3%	8%	26%	62%	3,5	0,61	389	1%
Dimensión Evaluación y retroalimentación a los alumnos	3%	10%	30%	57%	3,4	0,66	390	1%
Dimensión Relación con estudiantes	2%	4%	19%	76%	3,7	0,54	388	1%

(+) Para esta dimensión la escala de evaluación es la siguiente: Nunca o casi nunca (1), Solo en pocas clases (2), En varias clases (3), En la mayoría de las clases (4)

ENCUESTA DE CALIDAD DOCENTE

8. RELACIONES DE RESULTADOS CON CARACTERÍSTICAS DE LOS ALUMNOS

Para determinar cuán asociadas se encuentran dos medidas se utiliza la correlación. Esta puede variar desde un valor mínimo de -1 hasta un valor máximo de 1. Un valor de 0 indica que no existe relación. Un valor negativo indica una relación inversa, es decir, si aumenta una de las variables la otra disminuye. Por su parte, un valor positivo indica una relación directa, que ocurre cuando al aumentar una de las variables la otra también aumenta. Por ejemplo, si en la celda que cruza "Promedio de notas alumnos" con "Aplicación del conocimiento" apareciera 0,50, significaría que existe una tendencia a que los alumnos con mayor nota califiquen de forma más alta esa dimensión, y que los alumnos de menor nota la califiquen de forma más baja. NC indica que no se calculó la correlación debido a ausencia de varianza o a correlaciones con n<10.

Id1: 38538
ID: 16-11978406-KIN

	**Promedio notas alumnos**		**Asistencia reportada (+)**		**Dedicación semanal reportada (+)**	
	**Docente**	**UA**	**Docente**	**UA**	**Docente**	**UA**
Dimensión Aplicación del conocimiento	0,04	0,03	0,27*	0,1 *	0,07	-0,02
Dimensión Organización de la enseñanza	0,02	0,09	-0,09	0,07	0,15	0,03
Dimensión Metodologías enseñanza aprendizaje	-0,05	0,04	0,2	0,1 *	0,15	0,07
Dimensión Evaluación y retroalimentación a los alumnos	0,02	0,07	0,27*	0,13*	-0,01	0
Dimensión Relación con estudiantes	0,01	0,18*	-0,17	0,07	0,12	-0,07
Apreciación global (++)	0,05	0,04	-0,07	-0,01	0,2	-0,1
Aprendizaje percibido (+++)	-0,13	0,01	0,02	0,08	-0,07	0,01

* Indica que la correlación es estadísticamente significativa con $p<0.05$.

(+) Dato obtenido a partir del autorreporte de los alumnos en la encuesta.

(++) Solo con el propósito de relacionar el nivel de Apreciación global de los alumnos con notas, asistencia y dedicación de estos, se resumieron los puntajes de los 2 items de apreciación global en un solo puntaje.

(+++) Solo con el propósito de relacionar el nivel de Aprendizaje percibido de los alumnos con notas, asistencia y dedicación de estos, se resumieron los puntajes de los 2 items de aprendizaje percibido en un solo puntaje.

ENCUESTA DE CALIDAD DE LA DOCENCIA

REPORTE DE RESULTADOS PARA DOCENTES

Para este reporte se usaron datos extraídos entre el 18 y 22 de diciembre de 2014.

1. DATOS DE IDENTIFICACIÓN

Docente	[redacted]
UA del docente	
N° de docentes curso-secc.	
Tipo asignatura	
Sigla	
Sección - Total secciones	
Curso	
UA del curso	

2. DATOS DE LA EVALUACIÓN

Año y semestre aplicación	2014 - 22
Total de alumnos que completó la encuesta	43
Porcentaje encuestas respondidas en relación a alumnos inscritos	59%

3. CARACTERIZACIÓN ALUMNOS

Total de alumnos inscritos en el curso	73
Promedio Ponderado Acumulado (PPA) del grupo	5.3
Promedio de notas de los alumnos en el curso	5.1
Promedio de créditos aprobados por los alumnos *	192.8
Porcentaje alumnos que reportó que el curso contó con ayudantía presencial	59%

*Para su calculo se consideraron sólo promedios de créditos aprobados mayores a 0.

ENCUESTA DE CALIDAD DE LA DOCENCIA

Asistencia a clases - Dedicación fuera de clases - Carácter de inscripción del curso (declarado):

El porcentaje de clases (o actividades clínicas, terreno, laboratorio, etc.) a las que asistí en este curso con este profesor fue:					
**0% - 24%**	**25% - 49%**	**50% - 74%**	**75% - 100%**	**Total respuestas (n)**	**% omisión***
0%	0%	2%	98%	42	2%

¿aproximadamente cuántas horas dedicaste trabajando o estudiando para este curso fuera de las clases presenciales regulares?					
**3 o menos**	**4 a 6 horas**	**7 a 9 horas**	**10 o más**	**Total respuestas (n)**	**% omisión***
7%	37%	44%	12%	43	0%

Considerando la malla de tu carrera, indica en qué calidad tomaste este curso				
**Mínimo**	**Optativo de profundización**	**Optativo de formación general**	**Total respuestas (n)**	**% omisión***
93%	0%	7%	42	2%

*Corresponde al porcentaje de alumnos que no respondió el ítem.

ENCUESTA DE CALIDAD DE LA DOCENCIA

4. RESULTADOS DE ÍTEMS POR DIMENSIÓN

A continuación se presentan sus resultados por ítem agrupados según las dimensiones de esta evaluación.

- Porcentaje observado en cada opción de respuesta de cada ítem.
- Promedio de cada ítem.
- Desviación estándar de cada ítem. Informa la variabilidad de los puntajes de los participantes, es decir cuán cercanas o dispersas se encuentran las evaluaciones realizadas respecto del puntaje "promedio por ítem". Una desviación estándar de valor cero indicaría que todos los alumnos evaluaron a un profesor con un mismo puntaje; mientras más alto el valor del dato, mayor variabilidad de las respuestas de los estudiantes.
- El total de respuestas que se recibieron en cada ítem.
- El porcentaje de omisión por ítem, que corresponde a la proporción de estudiantes que respondieron la encuesta, pero no respondieron el ítem.

A nivel de cada dimensión reportada además se entrega la siguiente información:

- Porcentaje observado en cada opción de respuesta de los ítems que componen cada dimensión, en base al total de respuestas que recibió dicha dimensión.
- Promedio de cada dimensión, calculado en base al total de respuestas de los alumnos.
- Desviación estándar de cada dimensión, calculado en base al total de respuestas de los alumnos.
- El total de respuestas que se recibieron en cada dimensión.
- El porcentaje de omisión por dimensión, que corresponde a la proporción de estudiantes que respondió la encuesta pero no respondió ningún ítem de los que componen esa dimensión.

Las opciones de respuesta para los ítems corresponden a una escala de 4 puntos, siendo el valor mínimo 1 y el valor máximo 4, a excepción de las preguntas Sí – No del apartado de Apreciación Global (en que los valores máximos y mínimos son 1 y 0, respectivamente).

ENCUESTA DE CALIDAD DE LA DOCENCIA

4.1. Dimensión Aplicación del conocimiento

	Porcentaje de respuestas por ítem							
**¿Durante este curso (taller o laboratorio), con qué frecuencia el docente...**	**Nunca o casi nunca (1)**	**Solo en pocas clases (2)**	**En varias clases (3)**	**En la mayoría de las clases (4)**	**Prome-dio**	**Desv. están-dar**	**Total resptas. (n)**	**% omisión**
...usó bibliografía o recursos (de información, audiovisuales, artísticos u otros) variados y atingentes los objetivos del curso?	2%	2%	14%	81%	3.7	0.62	43	0%
... vinculó los contenidos del curso a ejemplos asociados a situaciones reales o hipotéticas?	0%	0%	5%	95%	4.0	0.21	43	0%
...presentó nuevas ideas, hallazgos o metodologías asociadas a los contenidos del curso?	0%	7%	19%	74%	3.7	0.61	42	2%
**Total dimensión Aplicación del conocimiento**	1%	3%	13%	84%	3.8	0.35	43	0%

ENCUESTA DE CALIDAD DE LA DOCENCIA

4.2. Dimensión Organización de la enseñanza

Con respecto a la organización de la enseñanza en este curso	Porcentaje de respuestas por ítem: Nunca o casi nunca (1)	Pocas veces (2)	Muchas veces (3)	Siempre o casi siempre (4)	Prome-dio	Desv. están-dar	Total resptas. (n)	% omisión
Los tiempos dedicados a las distintas actividades del curso fueron adecuados	0%	0%	28%	72%	3.7	0.45	43	0%
Existió una clara relación entre los contenidos del curso y las actividades pedagógicas implementadas (prácticas, laboratorios, reflexiones u otras)	0%	2%	12%	86%	3.8	0.44	42	2%
El desarrollo y secuencia de las clases, talleres o laboratorios fueron claras y facilitaron mi aprendizaje	0%	7%	12%	81%	3.7	0.58	43	0%
**Total de la dimensión Organización de la enseñanza**	0%	3%	17%	80%	3.8	0.35	43	0%

(este ítem no es considerado en el promedio de la dimensión)	Nunca o casi nunca (1)	Pocas veces (2)	Muchas veces (3)	Siempre o casi siempre (4)	Prome-dio	Desv. están-dar	Total resptas. (n)	% omisión
Hubo una adecuada coordinación de la ayudantía con el desarrollo del curso	0%	6%	13%	81%	3.7	0.58	31	28%

ENCUESTA DE CALIDAD DE LA DOCENCIA

4.3. Dimensión Metodología de enseñanza aprendizaje

	Porcentaje de respuestas por ítem							
**¿Durante este curso (taller o laboratorio), con qué frecuencia el docente estimuló...**	**Nunca o casi nunca (1)**	**Pocas veces (2)**	**Muchas veces (3)**	**Siempre o casi siempre (4)**	**Promedio**	**Desv. estándar**	**Total resptas. (n)**	**% omisión**
… que los estudiantes hicieran preguntas durante las clases?	2%	2%	12%	83%	3.8	0.62	42	2%
... que los estudiantes buscaran información o investigaran en forma autónoma?	2%	19%	26%	52%	3.3	0.86	42	2%
... que los estudiantes trabajaran en grupo en alguna actividad dentro o fuera de la sala de clases?	2%	5%	24%	68%	3.6	0.71	41	5%
**Total dimensión Metodología de enseñanza aprendizaje**	2%	9%	21%	68%	3.5	0.58	42	2%

4.4. Dimensión Evaluación y retroalimentación a los estudiantes

	Porcentaje de respuestas por ítem							
**¿Durante este curso (taller o laboratorio), con qué frecuencia el docente…**	**Nunca o casi nunca (1)**	**Pocas veces (2)**	**Muchas veces (3)**	**Siempre o casi siempre (4)**	**Promedio**	**Desv. estándar**	**Total resptas. (n)**	**% omisión**
… realizó evaluaciones con criterios explícitos y conocidos por todos los estudiantes?	0%	0%	29%	71%	3.7	0.46	42	2%
… entregó a tiempo retroalimentación (individual o grupal) acerca de las fortalezas y debilidades del desempeño de los estudiantes?	5%	5%	14%	76%	3.6	0.79	42	2%
… utilizó diferentes métodos, situaciones, formas o tipos de preguntas para evaluar los aprendizajes del curso?	2%	10%	21%	67%	3.5	0.77	42	2%
**Total dimensión Evaluación y retroalimentación a los estudiantes**	2%	5%	21%	71%	3.6	0.57	42	2%

ENCUESTA DE CALIDAD DE LA DOCENCIA

4.5. Dimensión Relación con estudiantes

Con respecto de la relación entre docente y estudiantes	Porcentaje de respuestas por ítem: Nunca o casi nunca (1)	Pocas veces (2)	Muchas veces (3)	Siempre o casi siempre (4)	Prome-dio	Desv. está-ndar	Total resptas. (n)	% omisión
El docente estuvo disponible para responder mis consultas en el horario acordado de atención de los estudiantes	2%	0%	14%	84%	3.8	0.56	43	0%
El docente fue respetuoso con todos los estudiantes del curso	0%	0%	7%	93%	3.9	0.26	43	0%
El docente mostró confianza en la capacidad de aprender de los estudiantes del curso	2%	0%	7%	91%	3.9	0.52	43	0%
**Total dimensión Relación con estudiantes**	2%	0%	9%	89%	3.9	0.42	43	0%

5. ÍTEMS DE APRECIACIÓN GLOBAL

En la siguiente tabla, el porcentaje de respuestas "Sí" corresponde a la proporción de estudiantes que respondieron la encuesta y marcaron esa opción. El porcentaje de omisión, es la proporción de estudiantes que respondieron la encuesta, pero no respondieron el ítem.

	% Sí	% No	Total respuestas (n)	% omisión
¿Recomendarías este profesor a un amigo?	95%	5%	43	0%
¿Quedaste satisfecho con este curso?	93%	7%	43	0%

ENCUESTA DE CALIDAD DE LA DOCENCIA

6. ÍTEMS DE APRENDIZAJE PERCIBIDO

	Porcentaje de respuestas por ítem							
	**Mucho menos de lo espera-do (1)**	**Menos de los espera-do (2)**	**Lo espera-do (3)**	**Mucho más de lo espera-do (4)**	**Prome-dio**	**Desv. están-dar**	**Total resptas. (n)**	**% omisión**
Más allá de la nota que has obtenido, ¿cuánto aprendiste en este curso?	2%	5%	51%	42%	3.3	0.68	43	0%
Este curso promovió mi pensamiento creativo, analítico o crítico	0%	2%	39%	59%	3.6	0.55	41	5%

7. RESUMEN RESULTADOS POR DIMENSIÓN: TOTAL UNIDAD ACADÉMICA

A continuación se presenta un resumen de los puntajes por dimensión para el Total Unidad Académica.

ID: 16-11978406-KIN Id1: 56018

	Porcentaje de respuestas por ítem							
	**Nunca o casi nunca (1)**	**Pocas veces (2)**	**Muchas veces (3)**	**Siempre o casi siempre (4)**	**Prome-dio**	**Desv. están-dar**	**Total resptas. (n)**	**% omisión**
Dimensión Aplicación del conocimiento (+)	1%	5%	20%	74%	3.7	0.51	575	0%
Dimensión Organización de la enseñanza	2%	6%	24%	68%	3.6	0.59	574	0%
Dimensión Metodologías enseñanza aprendizaje	2%	7%	23%	68%	3.6	0.56	567	1%
Dimensión Evaluación y retroalimentación a los alumnos	4%	12%	23%	61%	3.4	0.70	570	1%
Dimensión Relación con estudiantes	1%	4%	15%	81%	3.8	0.46	572	1%

(+) Para esta dimensión la escala de evaluación es la siguiente: Nunca o casi nunca (1), Solo en pocas clases (2), En varias clases (3), En la mayoría de las clases (4)
