# Supplementary material for: Local experience of laboratory activities in a BS physical therapy course: integrating sEMG and kinematics technology with active learning across six cohorts
Source: Front Neurol. 2024 Apr 25;15:1377222. doi: 10.3389/fneur.2024.1377222 (PMC11081031; doi:10.3389/fneur.2024.1377222)
Supplement: Supplementary file 2 [file Data_Sheet_2.PDF]

ENCUESTA DE CALIDAD DE LA DOCENCIA

8. RELACIONES DE RESULTADOS CON CARACTERÍSTICAS DE LOS ALUMNOS

Para determinar cuán asociadas se encuentran dos medidas se utiliza la correlación. Esta puede variar desde un valor mínimo de -1 hasta un valor máximo de 1. Un valor de 0 indica que no existe relación. Un valor negativo indica una relación inversa, es decir, si aumenta una de las variables la otra disminuye. Por su parte, un valor positivo indica una relación directa, que ocurre cuando al aumentar una de las variables la otra también aumenta. Por ejemplo, si en la celda que cruza "Promedio de notas alumnos" con "Aplicación del conocimiento" apareciera 0,50, significaría que existe una tendencia a que los alumnos con mayor nota califiquen de forma más alta esa dimensión, y que los alumnos de menor nota la califiquen de forma más baja. NC indica que no se calculó la correlación debido a ausencia de varianza o a correlaciones con n<10.

Id1: 56018
ID: 16-11978406-KIN

	**Promedio notas alumnos**		**Asistencia reportada (+)**		**Dedicación semanal reportada (+)**	
	**Docente**	**UA**	**Docente**	**UA**	**Docente**	**UA**
Dimensión Aplicación del conocimiento	0.11	0.1 *	0.14	0.11*	0.02	0.04
Dimensión Organización de la enseñanza	0.27	0.22*	0.13	0.13*	0.13	-0.02
Dimensión Metodologías enseñanza aprendizaje	-0.01	0.06	0.18	0.13*	0.04	0.1 *
Dimensión Evaluación y retroalimentación a los alumnos	0.19	0.17*	-0.13	0.16*	0.02	0.03
Dimensión Relación con estudiantes	0.08	0.15*	-0.07	0.14*	0.2	-0.1*
Apreciación global (++)	0.2	0.07	-0.05	0.14*	0.17	0.09*
Aprendizaje percibido (+++)	0.52 *	0.13*	0.01	0.19*	0.09	0.18*

* Indica que la correlación es estadísticamente significativa con $p<0.05$.

(+) Dato obtenido a partir del autorreporte de los alumnos en la encuesta.

(++) Solo con el propósito de relacionar el nivel de Apreciación global de los alumnos con notas, asistencia y dedicación de estos, se resumieron los puntajes de los 2 items de apreciación global en un solo puntaje.

(+++) Solo con el propósito de relacionar el nivel de Aprendizaje percibido de los alumnos con notas, asistencia y dedicación de estos, se resumieron los puntajes de los 2 items de aprendizaje percibido en un solo puntaje.

ENCUESTA DOCENTE
REPORTE DE RESULTADOS PARA DOCENTES

Para este reporte se utilizaron datos extraídos del sistema el día 17 de diciembre de 2015, a las 21:00 hrs.

DATOS DE IDENTIFICACIÓN

Docente	[redacted]
N° de docentes curso-secc.	
Tipo asignatura	
Sigla	
Sección - Total secciones	
Curso	
UA del curso	
Nombre UA del curso	

DATOS DE LA EVALUACIÓN

Año y semestre aplicación	2015-22
Total de alumnos que completó la encuesta	44
Porcentaje encuestas respondidas en relación a alumnos inscritos	59%

*Para su cálculo se consideraron sólo total de créditos aprobados mayores a 0

CARACTERIZACIÓN ALUMNOS

Total de alumnos inscritos en el curso	74
Promedio Global Acumulado (PGA) del grupo	5,2
Promedio de notas de los alumnos en el curso	5,4
Promedio de créditos aprobados por los alumnos*	197,4

RESUMEN RESULTADOS DEL DOCENTE

**(Promedio Frecuencia 1 a 4)**	**Docente**	**UA**	**PUC**
A) Aplicación del conocimiento (Preg. 1+2)	3,9	3,5	3,4
B) Organización de la enseñanza (Preg. 3)	3,8	3,4	3,2
C) Metodologías de Enseñanza Aprendizaje (Preg. 5)	3,8	3,6	3,4
D) Evaluación y Retroalimentación a los alumnos (Preg. 7+8)	3,7	3,4	3,3
E) Relación con los estudiantes (Preg. 9+10)	3,9	3,7	3,6

ENCUESTA DOCENTE
REPORTE DE RESULTADOS PARA DOCENTES

RESULTADOS

A) APLICACIÓN DEL CONOCIMIENTO	% Nunca o casi nunca (1)	% Pocas veces (2)	% Muchas veces (3)	% Siempre o casi siempre (4)	Promedio docente (1 a 4)	Desviación estándar	Total Respuestas (n)	% Omisión
1. ¿Con qué frecuencia el docente usó bibliografía o recursos atingentes a los objetivos del curso?	0%	2%	16%	82%	3,8	0,46	44	0,00%
2. ¿Con qué frecuencia el docente vinculó los contenidos del curso a ejemplos asociados a situaciones reales o hipotéticas?	0%	0%	2%	98%	4,0	0,15	44	0,00%
Total (Preg. 1+2)	0%	1%	9%	90%	3,9	0,26	44	0,00%

B) ORGANIZACIÓN DE LA ENSEÑANZA (*)	(1)	(2)	(3)	(4)	Promedio docente	Desviación estándar	Total Respuestas	% Omisión
3. El desarrollo y secuencia de las clases de este curso facilitó mi aprendizaje.	0%	2%	16%	82%	3,8	0,46	44	0,00%
4. Hubo una adecuada coordinación de la ayudantía con el desarrollo de las clases de este curso (% Omisión incluye No Aplica).	0%	10%	19%	71%	3,6	0,67	31	29,55%

(*) Las preguntas 3 y 4 miden aspectos de 'Organización de la Enseñanza', sin embargo, no existe un total agregado, ya que la pregunta 4 es respondida por una submuestra.

C) METODOLOGÍA DE ENSEÑANZA APRENDIZAJE (**)	(1)	(2)	(3)	(4)	Promedio docente	Desviación estándar	Total Respuestas	% Omisión
5. El docente estimuló que los estudiantes hicieran preguntas durante la clase.	0%	2%	18%	80%	3,8	0,48	44	0,00%
6. El docente promovió que los estudiantes buscaran información o investigaran en forma autónoma (% Omisión incluye No Aplica).	0%	2%	40%	58%	3,6	0,55	43	2,27%

(**) Las preguntas 5 y 6 miden aspectos de 'Metodología de Enseñanza Aprendizaje', sin embargo, no existe un total agregado, ya que la pregunta 6 es respondida por una submuestra.

ENCUESTA DOCENTE
REPORTE DE RESULTADOS PARA DOCENTES

D) EVALUACIÓN Y RETROALIMENTACIÓN A LOS ALUMNOS	% Nunca o casi nunca (1)	% Pocas veces (2)	% Muchas veces (3)	% Siempre o casi siempre (4)	Promedio docente	Desviación estándar	Total Respuestas (n)	% Omisión
7. El docente realizó evaluaciones con criterios explícitos y conocidos.	0%	0%	16%	84%	3,8	0,37	44	0,00%
8. El docente entregó a tiempo las notas de las evaluaciones.	0%	5%	30%	66%	3,6	0,58	44	0,00%
Total (Preg. 7+8)	0%	2%	23%	75%	3,7	0,41	44	0,00%

E) RELACIÓN CON LOS ESTUDIANTES	(1)	(2)	(3)	(4)	Promedio docente	Desviación estándar	Total Respuestas	% Omisión
9. El docente estuvo disponible para responder consultas de los estudiantes.	0%	0%	16%	84%	3,8	0,37	44	0,00%
10. El docente fue respetuoso con todos los estudiantes del curso.	0%	0%	2%	98%	4,0	0,15	44	0,00%
Total (Preg. 9+10)	0%	0%	9%	91%	3,9	0,22	44	0,00%

APRENDIZAJE PERCIBIDO	Mucho menos de lo esperado (1)	Menos de lo esperado (2)	Lo esperado (3)	Mucho más de lo esperado (4)	Promedio docente	Desviación estándar	Total Respuestas	% Omisión
11. Independiente de la nota que has obtenido, ¿cuánto aprendiste en este curso?	0%	5%	18%	77%	3,7	0,54	44	0,00%
12. Este curso promovió mi pensamiento creativo, analítico o crítico.	0%	0%	18%	82%	3,8	0,39	44	0,00%

ENCUESTA DOCENTE
REPORTE DE RESULTADOS PARA DOCENTES

DEDICACIÓN FUERA DE CLASES	% 3 horas o menos	% 4 a 6 horas	% 7 a 9 horas	% 10 o más horas	Total Respuestas	% Omisión
13. Sin considerar las clases presenciales, ¿cuántas horas dedicaste a este curso, en una semana promedio?	2%	32%	27%	39%	44	0,00%

APORTE DE ASISTENCIA AL CURSO	% Sí	% No	Total Respuestas	% Omisión
14. La asistencia a clases, ¿fue un aporte para comprender los contenidos del curso?	100%	0%	44	0,00%

APRECIACIÓN GLOBAL DEL CURSO	% Sí	% No	Total Respuestas	% Omisión
15. ¿Recomendarías este profesor a un amigo?	95%	5%	44	0,00%
16. ¿Quedaste satisfecho con este curso?	95%	5%	44	0,00%

RESUMEN DE RESULTADOS: TOTAL UNIDAD ACADÉMICA

A continuación se presenta un resumen para el Total Unidad Académica (UA)

PROMEDIO FRECUENCIA 1 A 4	% Nunca o casi nunca (1)	% Pocas veces (2)	% Muchas veces (3)	% Siempre o casi siempre (4)	Promedio UA (1 a 4)	Desviación estándar	Total Respuestas	% Omisión
A) Aplicación del conocimiento (Preg. 1+2)	2%	7%	25%	66%	3,5	0,57	994	0,00%
B) Organización de la enseñanza (Preg. 3)	2%	11%	30%	56%	3,4	0,78	992	0,20%
C) Metodologías de la Enseñanza Aprendizaje (Preg. 5)	1%	6%	26%	67%	3,6	0,65	993	0,10%
D) Evaluación y Retroalimentación a los alumnos (Preg. 7+8)	2%	11%	32%	55%	3,4	0,66	993	0,10%
E) Relación con los estudiantes (Preg. 9+10)	1%	4%	18%	78%	3,7	0,46	993	0,10%

ENCUESTA DOCENTE

REPORTE DE RESULTADOS PARA DOCENTES

Para este reporte se utilizaron datos extraídos del sistema el día 16/01/2017 a las 15:48:03

DATOS DE IDENTIFICACIÓN

Docente	[redacted]
N° de docentes curso sección	
Tipo de asignatura	
Créditos	
Sigla	
Sección-total secciones	
Nombre del curso	
UA del curso	
Nombre UA del curso	

DATOS DE LA EVALUACIÓN

Año y semestre aplicación	2016-22
Total de alumnos inscritos en el curso	101
Total de alumnos que completó la encuesta	42
Porcentaje encuestas respondidas en relación a alumnos inscritos	42,0%

CARACTERIZACIÓN DEL GRUPO CURSO

Promedio Global Acumulado (PGA) del grupo	5,2
Promedio de notas de los alumnos en el curso	5.8
Promedio de créditos aprobados por los alumnos*	204,8

*Para su cálculo se consideró sólo el total de créditos aprobados mayores a 0.

RESUMEN RESULTADOS

**(Promedio Frecuencia 1 a 4)**	DOCENTE	UNIDAD ACADÉMICA	UNIVERSIDAD
A) Aplicación del conocimiento (Preg.1+2)	3,9	3,6	3,4
B) Organización de la enseñanza (Preg.3)	3,9	3,4	3,2
C) Metodología de enseñanza aprendizaje (Preg.5)	3,8	3,6	3,5
D) Evaluación y retroalimentación a los estudiantes (Preg.7 + 8)	3,8	3,4	3,3
E) Relación con estudiantes (Preg.9+10)	3,9	3,7	3,6

ENCUESTA DOCENTE

REPORTE DE RESULTADOS PARA DOCENTES

RESULTADOS

A) APLICACIÓN DEL CONOCIMIENTO	% Nunca o casi nunca (1)	% Pocas veces (2)	% Muchas veces (3)	% Siempre o casi siempre (4)	Promedio Docente (1 a 4)	Desviación estándar	Total Respuestas (n)	% Omisión
1. ¿Con qué frecuencia el docente usó bibliografía o recursos atingentes a los objetivos del curso?	0%	0%	10%	90%	3,9	0,30	42	0,0%
2. ¿Con qué frecuencia el docente vinculó los contenidos del curso a ejemplos asociados a situaciones reales o hipotéticas?	0%	0%	5%	95%	4,0	0,22	42	0,0%
Total (Preg.1+2)	0%	0%	7%	93%	3,9	0,21	42	0,0%

B) ORGANIZACIÓN DE LA ENSEÑANZA (*)	% (1)	% (2)	% (3)	% (4)	Promedio Docente	Desviación estándar	Total Respuestas	% Omisión
3. El desarrollo y secuencia de las clases de este curso facilitó mi aprendizaje	0%	2%	7%	90%	3,9	0,40	42	0,0%
4. Hubo una adecuada coordinación de la ayudantía con el desarrollo de las clases de este curso (% Omisión incluye No Aplica)	14%	0%	0%	86%	3,6	1,13	7	83,3%

(*) Las preguntas 3 y 4 miden aspectos de "Organización de la enseñanza", sin embargo no existe un total agregado ya que la pregunta 4 es respondida por una sub muestra.

C) METODOLOGÍA DE ENSEÑANZA APRENDIZAJE (**)	% (1)	% (2)	% (3)	% (4)	Promedio Docente	Desviación estándar	Total Respuestas	% Omisión
5. El docente estimuló que los estudiantes hicieran preguntas durante las clases	0%	5%	12%	83%	3,8	0,52	42	0,0%
6. El docente promovió que los estudiantes buscaran información o investigaran en forma autónoma (% Omisión incluye No Aplica)	0%	0%	24%	76%	3,8	0,43	42	0,0%

(**) Las preguntas 5 y 6 miden aspectos de "Metodología de enseñanza aprendizaje", sin embargo no existe un total agregado ya que la pregunta 6 es respondida por una sub muestra.

ENCUESTA DOCENTE

REPORTE DE RESULTADOS PARA DOCENTES

D) EVALUACIÓN Y RETROALIMENTACIÓN A LOS ESTUDIANTES	% Nunca o casi nunca (1)	% Pocas veces (2)	% Muchas veces (3)	% Siempre o casi siempre (4)	Promedio Docente (1 a 4)	Desviación estándar	Total Respuestas (n)	% Omisión
7. El docente realizó evaluaciones con criterios explícitos y conocidos	0%	0%	12%	88%	3,9	0,33	42	0,0%
8. El docente entregó a tiempo las notas de las evaluaciones	2%	5%	17%	76%	3,7	0,69	42	0,0%
Total (Preg.7+8)	1%	2%	14%	82%	3,8	0,43	42	0,0%

E) RELACIÓN CON LOS ESTUDIANTES	% (1)	% (2)	% (3)	% (4)	Promedio Docente	Desviación estándar	Total Respuestas	% Omisión
9. El docente estuvo disponible para responder consultas de los estudiantes	0%	2%	10%	88%	3,9	0,42	42	0,0%
10. El docente fue respetuoso con todos los estudiantes del curso	0%	0%	5%	95%	4,0	0,22	42	0,0%
Total (Preg.9+10)	0%	1%	7%	92%	3,9	0,25	42	0,0%

F) APRENDIZAJE	% Mucho menos de lo esperado (1)	% Menos de lo esperado (2)	% Lo esperado (3)	% Mucho más de lo esperado (4)	Promedio Docente (1 a 4)	Desviación estándar	Total Respuestas (n)	% Omisión
11. Independiente de la nota que has obtenido, ¿cuánto aprendiste en este curso?	0%	0%	29%	71%	3,7	0,46	42	0,0%
12. Este curso promovió mi pensamiento creativo, analítico o crítico	0%	0%	21%	79%	3,8	0,42	42	0,0%

ENCUESTA DOCENTE

REPORTE DE RESULTADOS PARA DOCENTES

G) DEDICACIÓN FUERA DE CLASES	% 3 horas o menos	% 4 a 6 horas	% 7 a 9 horas	% 10 o más horas	Total Respuestas	% Omisión
13. Sin considerar las clases presenciales, ¿cuántas horas dedicaste a este curso, en una semana promedio?	2%	29%	45%	24%	42	0,0%

H) APORTE DE LA ASISTENCIA A CLASES	% Sí	% No	Total Respuestas	% Omisión
14. La asistencia a clases, ¿fue un aporte para comprender los contenidos del curso?	100%	0%	42	0,0%

I) APRECIACIÓN GLOBAL DEL CURSO	% Sí	% No	Total Respuestas	% Omisión
15. ¿Recomendarías este profesor a un amigo?	100%	0%	42	0,0%
16. ¿Quedaste satisfecho con este curso?	98%	2%	42	0,0%

RESUMEN DE RESULTADOS: TOTAL UNIDAD ACADÉMICA

A continuación se presenta un resumen para el Total Unidad Académica (UA).

	% Nunca o casi nunca (1)	% Pocas veces (2)	% Muchas veces (3)	% Siempre o casi siempre (4)	Promedio UA (1 a 4)	Desviación estándar	Total Respuestas (n)	% Omisión
A) Aplicación del conocimiento (Preg.1+2)	2%	6%	21%	71%	3,6	0,58	1002	0,0%
B) Organización de la enseñanza (Preg.3)	3%	10%	29%	58%	3,4	0,78	1002	0,0%
C) Metodología de enseñanza aprendizaje (Preg.5)	1%	6%	20%	72%	3,6	0,65	1000	0,2%
D) Evaluación y retroalimentación a los estudiantes (Preg.7 + 8)	4%	9%	27%	60%	3,4	0,68	1001	0,1%
E) Relación con los estudiantes (Preg.9+10)	1%	4%	17%	78%	3,7	0,49	1001	0,1%

ENCUESTA DOCENTE

REPORTE DE RESULTADOS PARA DOCENTES

Para este reporte se utilizaron datos extraídos del sistema el día 02/01/2018 a las 12:39:41

DATOS DE IDENTIFICACIÓN

Docente	[redacted]
N° de docentes curso sección	
Tipo de asignatura	
Créditos	
Sigla	
Sección-total secciones	
Nombre del curso	
UA del curso	
Nombre UA del curso	

DATOS DE LA EVALUACIÓN

Año y semestre aplicación	2017-22
Total de alumnos inscritos en el curso	72
Total de alumnos que completó la encuesta	51
Porcentaje encuestas respondidas en relación a alumnos inscritos	71,0%

CARACTERIZACIÓN DEL GRUPO CURSO

Promedio Global Acumulado (PGA) del grupo	5,3
Promedio de notas de los alumnos en el curso	5.7
Promedio de créditos aprobados por los alumnos*	211,4

*Para su cálculo se consideró sólo el total de créditos aprobados mayores a 0.

RESUMEN RESULTADOS

**(Promedio Frecuencia 1 a 4)**	DOCENTE	UNIDAD ACADÉMICA	UNIVERSIDAD
A) Aplicación del conocimiento (Preg.1+2)	3,9	3,7	3,5
B) Organización de la enseñanza (Preg.3)	3,8	3,5	3,3
C) Metodología de enseñanza aprendizaje (Preg.5)	3,8	3,7	3,5
D) Evaluación y retroalimentación a los estudiantes (Preg.7 + 8)	3,8	3,5	3,4
E) Relación con estudiantes (Preg.9+10)	3,9	3,8	3,7

ENCUESTA DOCENTE

REPORTE DE RESULTADOS PARA DOCENTES

RESULTADOS

A) APLICACIÓN DEL CONOCIMIENTO	% Nunca o casi nunca (1)	% Pocas veces (2)	% Muchas veces (3)	% Siempre o casi siempre (4)	Promedio Docente (1 a 4)	Desviación estándar	Total Respuestas (n)	% Omisión
1. ¿Con qué frecuencia el docente usó bibliografía o recursos atingentes a los objetivos del curso?	0%	0%	6%	94%	3,9	0,24	51	0,0%
2. ¿Con qué frecuencia el docente vinculó los contenidos del curso a ejemplos asociados a situaciones reales o hipotéticas?	0%	0%	6%	94%	3,9	0,24	51	0,0%
Total (Preg.1+2)	0%	0%	6%	94%	3,9	0,19	51	0,0%

B) ORGANIZACIÓN DE LA ENSEÑANZA (*)	% (1)	% (2)	% (3)	% (4)	Promedio Docente	Desviación estándar	Total Respuestas	% Omisión
3. El desarrollo y secuencia de las clases de este curso facilitó mi aprendizaje	0%	0%	20%	80%	3,8	0,40	51	0,0%
4. Hubo una adecuada coordinación de la ayudantía con el desarrollo de las clases de este curso (% Omisión incluye No Aplica)	0%	0%	9%	91%	3,9	0,30	11	78,4%

(*) Las preguntas 3 y 4 miden aspectos de "Organización de la enseñanza", sin embargo no existe un total agregado ya que la pregunta 4 es respondida por una sub muestra.

C) METODOLOGÍA DE ENSEÑANZA APRENDIZAJE (**)	% (1)	% (2)	% (3)	% (4)	Promedio Docente	Desviación estándar	Total Respuestas	% Omisión
5. El docente estimuló que los estudiantes hicieran preguntas durante las clases	0%	0%	16%	84%	3,8	0,37	51	0,0%
6. El docente promovió que los estudiantes buscaran información o investigaran en forma autónoma (% Omisión incluye No Aplica)	0%	6%	16%	78%	3,7	0,58	49	3,9%

(**) Las preguntas 5 y 6 miden aspectos de "Metodología de enseñanza aprendizaje", sin embargo no existe un total agregado ya que la pregunta 6 es respondida por una sub muestra.

ENCUESTA DOCENTE

REPORTE DE RESULTADOS PARA DOCENTES

D) EVALUACIÓN Y RETROALIMENTACIÓN A LOS ESTUDIANTES	% Nunca o casi nunca (1)	% Pocas veces (2)	% Muchas veces (3)	% Siempre o casi siempre (4)	Promedio Docente (1 a 4)	Desviación estándar	Total Respuestas (n)	% Omisión
7. El docente realizó evaluaciones con criterios explícitos y conocidos	0%	0%	8%	92%	3,9	0,27	51	0,0%
8. El docente entregó a tiempo las notas de las evaluaciones	0%	4%	33%	63%	3,6	0,57	51	0,0%
Total (Preg.7+8)	0%	2%	21%	77%	3,8	0,35	51	0,0%

E) RELACIÓN CON LOS ESTUDIANTES	% (1)	% (2)	% (3)	% (4)	Promedio Docente	Desviación estándar	Total Respuestas	% Omisión
9. El docente estuvo disponible para responder consultas de los estudiantes	0%	0%	12%	88%	3,9	0,33	51	0,0%
10. El docente fue respetuoso con todos los estudiantes del curso	0%	0%	2%	98%	4,0	0,14	51	0,0%
Total (Preg.9+10)	0%	0%	7%	93%	3,9	0,17	51	0,0%

F) APRENDIZAJE	% Mucho menos de lo esperado (1)	% Menos de lo esperado (2)	% Lo esperado (3)	% Mucho más de lo esperado (4)	Promedio Docente (1 a 4)	Desviación estándar	Total Respuestas (n)	% Omisión
11. Independiente de la nota que has obtenido, ¿cuánto aprendiste en este curso?	0%	0%	22%	78%	3,8	0,42	51	0,0%
12. Este curso promovió mi pensamiento creativo, analítico o crítico	0%	0%	18%	82%	3,8	0,39	51	0,0%

ENCUESTA DOCENTE

REPORTE DE RESULTADOS PARA DOCENTES

G) DEDICACIÓN FUERA DE CLASES	% 3 horas o menos	% 4 a 6 horas	% 7 a 9 horas	% 10 o más horas	Total Respuestas	% Omisión
13. Sin considerar las clases presenciales, ¿cuántas horas dedicaste a este curso, en una semana promedio?	8%	45%	27%	20%	51	0,0%

H) APORTE DE LA ASISTENCIA A CLASES	% Sí	% No	Total Respuestas	% Omisión
14. La asistencia a clases, ¿fue un aporte para comprender los contenidos del curso?	100%	0%	51	0,0%

I) APRECIACIÓN GLOBAL DEL CURSO	% Sí	% No	Total Respuestas	% Omisión
15. ¿Recomendarías este profesor a un amigo?	100%	0%	51	0,0%
16. ¿Quedaste satisfecho con este curso?	98%	2%	51	0,0%

RESUMEN DE RESULTADOS: TOTAL UNIDAD ACADÉMICA

A continuación se presenta un resumen para el Total Unidad Académica (UA).

	% Nunca o casi nunca (1)	% Pocas veces (2)	% Muchas veces (3)	% Siempre o casi siempre (4)	Promedio UA (1 a 4)	Desviación estándar	Total Respuestas (n)	% Omisión
A) Aplicación del conocimiento (Preg.1+2)	2%	4%	17%	77%	3,7	0,58	1202	0,0%
B) Organización de la enseñanza (Preg.3)	3%	8%	24%	65%	3,5	0,77	1202	0,0%
C) Metodología de enseñanza aprendizaje (Preg.5)	2%	4%	19%	76%	3,7	0,62	1201	0,1%
D) Evaluación y retroalimentación a los estudiantes (Preg.7 + 8)	2%	8%	24%	66%	3,5	0,65	1201	0,1%
E) Relación con los estudiantes (Preg.9+10)	1%	4%	13%	82%	3,8	0,53	1201	0,1%

ENCUESTA DOCENTE

REPORTE DE RESULTADOS PARA DOCENTES

Para este reporte se utilizaron datos extraídos del sistema el día 02/01/2019 a las 09:39:52

DATOS DE IDENTIFICACIÓN

Docente	[redacted]
N° de docentes curso sección	
Tipo de asignatura	
Créditos	
Sigla	
Sección-total secciones	
Nombre del curso	
UA del curso	
Nombre UA del curso	

DATOS DE LA EVALUACIÓN

Año y semestre aplicación	2018-22
Total de alumnos inscritos en el curso	95
Total de alumnos que completó la encuesta	61
Porcentaje encuestas respondidas en relación a alumnos inscritos	64,0%

CARACTERIZACIÓN DEL GRUPO CURSO

Promedio Global Acumulado (PGA) del grupo	5,3
Promedio de notas de los alumnos en el curso	5.6
Promedio de créditos aprobados por los alumnos*	206,3

*Para su cálculo se consideró sólo el total de créditos aprobados mayores a 0.

RESUMEN RESULTADOS

**(Promedio Frecuencia 1 a 4)**	DOCENTE	UNIDAD ACADÉMICA	UNIVERSIDAD
A) Aplicación del conocimiento (Preg.1+2)	3,9	3,7	3,5
B) Organización de la enseñanza (Preg.3)	3,7	3,5	3,3
C) Metodología de enseñanza aprendizaje (Preg.5)	3,9	3,8	3,5
D) Evaluación y retroalimentación a los estudiantes (Preg.7 + 8)	3,6	3,5	3,4
E) Relación con estudiantes (Preg.9+10)	3,9	3,8	3,7

ENCUESTA DOCENTE

REPORTE DE RESULTADOS PARA DOCENTES

RESULTADOS

A) APLICACIÓN DEL CONOCIMIENTO	% Nunca o casi nunca (1)	% Pocas veces (2)	% Muchas veces (3)	% Siempre o casi siempre (4)	Promedio Docente (1 a 4)	Desviación estándar	Total Respuestas (n)	% Omisión
1. ¿Con qué frecuencia el docente usó bibliografía o recursos atingentes a los objetivos del curso?	0%	0%	10%	90%	3,9	0,30	61	0,0%
2. ¿Con qué frecuencia el docente vinculó los contenidos del curso a ejemplos asociados a situaciones reales o hipotéticas?	0%	0%	7%	93%	3,9	0,25	61	0,0%
Total (Preg.1+2)	0%	0%	8%	92%	3,9	0,23	61	0,0%

B) ORGANIZACIÓN DE LA ENSEÑANZA (*)	% (1)	% (2)	% (3)	% (4)	Promedio Docente	Desviación estándar	Total Respuestas	% Omisión
3. El desarrollo y secuencia de las clases de este curso facilitó mi aprendizaje	0%	2%	23%	75%	3,7	0,48	61	0,0%
4. Hubo una adecuada coordinación de la ayudantía con el desarrollo de las clases de este curso (% Omisión incluye No Aplica)	0%	2%	24%	75%	3,7	0,49	55	9,8%

(*) Las preguntas 3 y 4 miden aspectos de "Organización de la enseñanza", sin embargo no existe un total agregado ya que la pregunta 4 es respondida por una sub muestra.

C) METODOLOGÍA DE ENSEÑANZA APRENDIZAJE (**)	% (1)	% (2)	% (3)	% (4)	Promedio Docente	Desviación estándar	Total Respuestas	% Omisión
5. El docente estimuló que los estudiantes hicieran preguntas durante las clases	0%	0%	10%	90%	3,9	0,30	61	0,0%
6. El docente promovió que los estudiantes buscaran información o investigaran en forma autónoma (% Omisión incluye No Aplica)	0%	3%	15%	82%	3,8	0,49	61	0,0%

(**) Las preguntas 5 y 6 miden aspectos de "Metodología de enseñanza aprendizaje", sin embargo no existe un total agregado ya que la pregunta 6 es respondida por una sub muestra.

ENCUESTA DOCENTE

REPORTE DE RESULTADOS PARA DOCENTES

D) EVALUACIÓN Y RETROALIMENTACIÓN A LOS ESTUDIANTES	% Nunca o casi nunca (1)	% Pocas veces (2)	% Muchas veces (3)	% Siempre o casi siempre (4)	Promedio Docente (1 a 4)	Desviación estándar	Total Respuestas (n)	% Omisión
7. El docente realizó evaluaciones con criterios explícitos y conocidos	0%	0%	10%	90%	3,9	0,30	61	0,0%
8. El docente entregó a tiempo las notas de las evaluaciones	3%	18%	33%	46%	3,2	0,86	61	0,0%
Total (Preg.7+8)	2%	9%	21%	68%	3,6	0,47	61	0,0%

E) RELACIÓN CON LOS ESTUDIANTES	% (1)	% (2)	% (3)	% (4)	Promedio Docente	Desviación estándar	Total Respuestas	% Omisión
9. El docente estuvo disponible para responder consultas de los estudiantes	0%	0%	10%	90%	3,9	0,30	61	0,0%
10. El docente fue respetuoso con todos los estudiantes del curso	0%	0%	5%	95%	4,0	0,22	61	0,0%
Total (Preg.9+10)	0%	0%	7%	93%	3,9	0,22	61	0,0%

F) APRENDIZAJE	% Mucho menos de lo esperado (1)	% Menos de lo esperado (2)	% Lo esperado (3)	% Mucho más de lo esperado (4)	Promedio Docente (1 a 4)	Desviación estándar	Total Respuestas (n)	% Omisión
11. Independiente de la nota que has obtenido, ¿cuánto aprendiste en este curso?	0%	2%	34%	64%	3,6	0,52	61	0,0%
12. Este curso promovió mi pensamiento creativo, analítico o crítico	0%	3%	23%	74%	3,7	0,53	61	0,0%

ENCUESTA DOCENTE

REPORTE DE RESULTADOS PARA DOCENTES

G) DEDICACIÓN FUERA DE CLASES	% 3 horas o menos	% 4 a 6 horas	% 7 a 9 horas	% 10 o más horas	Total Respuestas	% Omisión
13. Sin considerar las clases presenciales, ¿cuántas horas dedicaste a este curso, en una semana promedio?	13%	36%	41%	10%	61	0,0%

H) APORTE DE LA ASISTENCIA A CLASES	% Sí	% No	Total Respuestas	% Omisión
14. La asistencia a clases, ¿fue un aporte para comprender los contenidos del curso?	95%	5%	61	0,0%

I) APRECIACIÓN GLOBAL DEL CURSO	% Sí	% No	Total Respuestas	% Omisión
15. ¿Recomendarías este profesor a un amigo?	98%	2%	61	0,0%
16. ¿Quedaste satisfecho con este curso?	98%	2%	61	0,0%

RESUMEN DE RESULTADOS: TOTAL UNIDAD ACADÉMICA

A continuación se presenta un resumen para el Total Unidad Académica (UA).

	% Nunca o casi nunca (1)	% Pocas veces (2)	% Muchas veces (3)	% Siempre o casi siempre (4)	Promedio UA (1 a 4)	Desviación estándar	Total Respuestas (n)	% Omisión
A) Aplicación del conocimiento (Preg.1+2)	1%	4%	17%	79%	3,7	0,45	1061	0,0%
B) Organización de la enseñanza (Preg.3)	2%	7%	28%	64%	3,5	0,69	1061	0,0%
C) Metodología de enseñanza aprendizaje (Preg.5)	1%	3%	16%	80%	3,8	0,53	1061	0,0%
D) Evaluación y retroalimentación a los estudiantes (Preg.7 + 8)	2%	8%	25%	65%	3,5	0,60	1061	0,0%
E) Relación con los estudiantes (Preg.9+10)	1%	2%	12%	85%	3,8	0,40	1061	0,0%
