# Supplementary material for: Local experience of laboratory activities in a BS physical therapy course: integrating sEMG and kinematics technology with active learning across six cohorts
Source: Front Neurol. 2024 Apr 25;15:1377222. doi: 10.3389/fneur.2024.1377222 (PMC11081031; doi:10.3389/fneur.2024.1377222)
Supplement: Supplementary file 3 [file Data_Sheet_3.PDF]

Quiz example for "Shoulder ascending and descending cycle Laboratory."

Instruction: All questions are extracted from the obligatory lectures "Shoulder Complex and Elbow chapter of Kynesiology of Musculoskeletal system text" and "J Electromyogr Kinesiol. 2010;20(2):212-22 of Wickham," which was given at the beginning the semester. You have 12 minutes to answer seven multiple-choice questions. Only one is correct, and the wrong answer is not discounted from the correct answers. Only one clearly circle-marked answer will be accepted. In case of a mistake, please cross out all the wrong answers with a line. To pass the quiz, you must score a minimum of 4 correct answers (60% of difficulty).

Name:…….

Date: ………………./……………………………../……………………………………………………………………………….

Course name:………

QUESTIONS

1.- According to Wickham's paper. Which is the first shoulder muscle to activate (average onset) during the shoulder abduction?. The ____________ muscle.

- **a. Upper Trapezius**
- **b. Lower Trapezius**
- **c. Suprascapularis**
- **d. Supraspinatus**

2.- According to Neuman's text. Which scapular movement accompanies the shoulder abduction?. The __________ of the scapula.

- **a. Lateral or upward Rotation**
- **b. Descending**
- **c. Approximation**
- **d. No movement**

3.- According to Wickham's paper. Which is the abduction range of motion where more shoulder muscle reaches their "peak intensity"?. Is it between ___________.:

- **a. 0º and 60º**
- **b. 90º and 140º**
- **c. 60º and 90º**
- **d. 140º and 180º**

4.- According to Wickham's paper. For what purpose can the sEMG be used?. To measure muscle ____________.:

- **a. Activation**
- **b. Force**
- **c. Energy**
- **d. Work**

5.- According to Neuman's text. What translation movement would the biceps brachialis cause on the head of the humerus when activated?. The___________of the humerus head.:

- **a. Rotation toward lateral**
- **b. Ascent**
- **c. Descent**
- **d. Rotation toward medial**

6.- According to Neuman's text. What shoulder movement in their craniocaudal axis is necessary to avoid excessive subacromial bursa compression?. The __________ movement.:

- **a. Lateral Rotation**
- **b. Medial Rotation**
- **c. Upward Rotation**
- **d. No movement is requiered**

7.- Considering the action of the scapula during the shoulder abduction described by Neuman's text and the activation information given by Wickham's paper. What kind of action is likely performed on the middle trapezius muscle during shoulder abduction? A/AN ____________action to control the lateral or upward Rotation of the scapula.:

- **a. Concentric**
- **b. Anisometric**
- **c. Isometric**
- **d. Eccentric**
