# Supplementary material for: Local experience of laboratory activities in a BS physical therapy course: integrating sEMG and kinematics technology with active learning across six cohorts
Source: Front Neurol. 2024 Apr 25;15:1377222. doi: 10.3389/fneur.2024.1377222 (PMC11081031; doi:10.3389/fneur.2024.1377222)
Supplement: Supplementary file 4 [file Data_Sheet_4.pdf]

Motor tasks

Seven motor tasks were considered in the activities based on a problem-based approach and real-life problems:

Problem 1: How do the leg muscles work to avoid a fall when the bipedal posture is perturbed? Three experiments were developed to answer this question. The first experiment involved a non-expected anteroposterior perturbation applied to a volunteer standing with eyes closed on a force plate (1). The motor control strategies were quantified using an accelerometer placed on the sternum region and sEMG sensors attached to the tibialis anterior and medial gastrocnemius muscles (1). The second experiment involved the same anteroposterior perturbation while standing with eyes open. In the third experiment, the volunteer maintained a standing posture with elbows extended, forming 90º of shoulder flexion and a neutral wrist position and holding a cord connected to weight (4.5 kg). Then, an expected perturbation was produced when the professor suddenly cut the cord, changing the muscles' external torque to control the posture (1). Obligatory readings: Chapters addressing the voluntary control of a single muscle and postural control from Neurophysiological Basis of Movement by Mark L. Latash, De Luca et al., J Appl Biomech. 1993: 13(2): 135-163, and Laboratory guide instructions (Table 1).

Problem 2: How do the muscles control and stabilize the shoulder joint complex during upper limb movements in the scapular plane? Two experiments were developed to answer this question. The first experiment involved a volunteer standing upright and requesting a shoulder abduction. The motor control strategies were quantified using a rigid, passive kinematic marker attached to the wrist, and a sEMG sensor was placed over the medial deltoids, serratus anterioris, upper, middle, and inferior trapezius muscles (Figure 2). The second experiment involved the same volunteer holding a dumbbell of 6 kg and being requested to perform a shoulder abduction against the external load. Obligatory readings: Chapters addressing the Shoulder Complex and Elbow from Kinesiology of the Musculoskeletal System: Foundations for Rehabilitation by Donald A. Neumann, Wickham et al., J Electromyogr Kinesiol. 2010;20(2):212-22, and Laboratory guide instructions (Table 1).

Problem 3: How do the upper limb and arm muscles control and stabilize the wrist joint during the maximal handgrip in extension, neutral, and flexion positions? Three experiments were developed to answer this question. The first experiment involved a volunteer performing the maximal handgrip strength in a neutral wrist position using sEMG sensors attached to the flexor and extensor wrist muscles and biceps and triceps brachialis. The second experiment involved the same volunteer with attached sensors performing a maximal handgrip while the wrist was in maximal extension. The third experiment involved the same volunteer with the same attached sensors performing a maximal handgrip while the wrist was in maximal flexion. Obligatory readings: Chapter addressing Elbow and forearm, wrist, and hand from Kinesiology of the Musculoskeletal System: Foundations for Rehabilitation by Donald A. Neumann, Di Domizio, et al., Ergonomics. 2010;53(3):336-43, and Laboratory guide instructions (Table 1).

Problem 4: How do the trunk muscles control and stabilize the spine during lifting? Two experiments were developed to answer this question. The first experiment

involved a volunteer lifting a weight of 9 kg with hip flexion and knee extension. At the same time, sEMG sensors were placed on the multifidus, iliocostalis, rectus abdominis, and external oblique muscles. The second experiment involved a volunteer lifting a weight of 9 kg with hip and knee flexion using the same attached sensors. Obligatory readings: Chapter addressing the axial skeleton from Kinesiology of the Musculoskeletal System: Foundations for Rehabilitation by Donald A. Neumann, Gupta, et al., J Biomech. 2001;34(4):491-6, Holm et al., J Electromyogr Kinesiol. 2002 Jun;12(3):219-34, and Laboratory guide instructions (Table 1).

Problem 5: How do the hip muscles control and stabilize the hip joint during walking? One experiment was developed to answer this question. The experiment involved a volunteer walking over two force platforms at self-selected gait speed in a straight line for 5 m, without obstacles, and kinematic markers were attached to the lower limbs to record movement. In contrast, sEMG sensors were attached to rectus femoris, gluteus maximum, and medius to record muscle activation. Obligatory readings: Chapter addressing the Hip and Kinesiology of walking from Kinesiology of the Musculoskeletal System: Foundations for Rehabilitation by Donald A. Neumann, Sutherland et al., Gait Posture. 2001;14(1):61-70, Sutherland et al., Gait Posture. 2002;16(2):159-79, and Laboratory guide instructions (Table 1).

Problem 6: How do the knee muscles control and stabilize the knee joint during walking? One experiment was developed to answer this question. The experiment involved a volunteer walking over two force platforms at self-selected gait speed in a straight line for 5 m, without obstacles, using kinematic 14 mm spherical landmarks attached to lower limbs to record movement and sEMG sensor attached to rectus femoris, vastus medialis, biceps femoralis, and semitendinosus to record muscle activation. Obligatory readings: Chapters addressing the Knee and Kinesiology of walking from Kinesiology of the Musculoskeletal System: Foundations for Rehabilitation by Donald A. Neumann, Sutherland et al., Gait Posture. 2001;14(1):61-70, Sutherland et al., Gait Posture. 2002;16(2):159-79, and Laboratory guide instructions (Table 1).

Problem 7: How do the ankle muscles control and stabilize the ankle joint during walking? One experiment was developed to answer this question. The experiment involved a volunteer walking over two force platforms at self-selected gait speed in a straight line for 5 m, without obstacles, using kinematic markers attached to lower limbs to record movement and sEMG sensor attached to tibialis anterior, peroneus longus, and gastrocnemius medialis to record muscle activation. Obligatory readings: Chapters addressing the Ankle and Kinesiology of walking from Kinesiology of the Musculoskeletal System: Foundations for Rehabilitation by Donald A. Neumann, Sutherland et al., Gait Posture. 2001;14(1):61-70, Sutherland et al., Gait Posture. 2002;16(2):159-79, and Laboratory guide instructions (Table 1).

Instrumentation

Depending on the laboratory activity, Kinematics variables were measured with isoinertial accelerometers or 3D motion capture. The raw accelerometry signals were collected using a Trigno^{TM} Wireless 16-Channel accelerometry system (Delsys, Inc., Boston, USA) sampling data at 148.15 Hz. The sensors were of 3 degrees of freedom, range of ±3g, Bandwidth of 24 Hz – 470 Hz, basal noise (RMS) of 0.016g, offset error of ± 0.21 g for the XY axis and -0.42 g for the Z-axis, and resolution of 10 bits. The 3D

motion used the lower limb plug-in-gait model with 8-infrared Bonita cameras (Vicon Motion Systems Inc., Oxford, UK), sampling data at 100 Hz through Nexus 1.8.5 (Vicon Motion Systems Ltd., UK). A low pass cut-off frequency of 6 Hz was used for offline processing with a second-order Butterworth filter.

Neurophysiological measurements considered the neuromuscular electrical activation specified in the motor task section. The raw EMG signals were collected using a Trigno™ Wireless 16-Channel EMG system (Delsys Inc., Boston, USA) with a bar parallel shape silver–silver chloride material, and an inter-electrode distance of 10 mm, CMRR > 80 dB, a gain of 1000, analog bandpass filtered at 20–450 Hz. Signals were sampled at 2000 Hz through Nexus 1.8.5 (Vicon Motion Systems Inc., Oxford, UK). The EMG signals were mean-centered and filtered with a bandpass cut-off frequency of 20–450 Hz with a second-order Butterworth filter. The magnitude of neuromuscular electrical activation was plotted with different methods: The first laboratory used the raw signals, the second laboratory used a full-rectification of the sEMG (Figure 2), the third laboratory used a root mean square envelope with a window length of 250 ms and sliding of 1 sample, the fourth laboratory used both raw signals and full rectified signals, the fifth to the seventh laboratory used both raw and envelop signals obtained from low-pass filter of 30 Hz with a second-order Butterworth filter. Some laboratories included the sEMG normalization regarding the maximal voluntary contraction to show the student their differences concerning not normalizing. When the timing was the center of the analysis, the raw signal was mainly used. The raw signals and envelopes were used when activation patterns were the center of the analysis. When the signal intensity was mainly discussed, the signals were full-rectified and normalized, or a normalized root mean square envelope was used. Different methods had the didactic aim of introducing the relevance of these procedures in sEMG for PT students. All signal processing was conducted using custom-made codes prepared for the laboratory activities.

Electrodes and anatomical references were placed on a volunteer by the students with the support of the faculties. The SENIAM guideline was used for sEMG placing and skin preparation protocols to determine the availability of the recommendations at this date (2). The faculties trained the students in a previous meeting (Figure 1B), and they studied the SENIAM guidelines necessary for their activity prior to the laboratory, which were also projected as slides and revised *in vivo* with all the participants. The faculties placed the kinematic markers (rigid, passive, and spherical markers of 14 mm diameter of B&L ENGINEERING®) or accelerometers because kinematics recordings require more expertise in finding the joint centers, and it was decided that the students focused on sEMG collection rather than kinematics by time assumptions.

References

1. Latash ML. *Neurophysiological Basis of Movement*. Human Kinetics (2008). 444 p.

2. Hermens HJ, Freriks B, Disselhorst-Klug C, Rau G. Development of recommendations for SEMG sensors and sensor placement procedures. *J Electromyogr Kinesiol* (2000) 10:361–374. doi: 10.1016/s1050-6411(00)00027-4
